# Exploring the Tiers of Rooted Phylogenetic Network Space Using Tail Moves

**DOI:** 10.1007/s11538-018-0452-0

**Published:** 2018-06-14

**Authors:** Remie Janssen, Mark Jones, Péter L. Erdős, Leo van Iersel, Celine Scornavacca

**Affiliations:** 1 Delft Institute of Applied Mathematics, Delft University of Technology, Postbus 5031, 2600 GA Delft, The Netherlands; 2 MTA Rényi Institute of Mathematics, Reáltanoda u 13-15, Budapest, 1053 Hungary; 3 Institut des Sciences de l’Evolution, CNRS, IRD, EPHE, Université de Montpellier, Place Eugène Bataillon, Montpellier, France; 4 Institut de Biologie Computationnelle (IBC), Place Eugène Bataillon, Montpellier, France

**Keywords:** Phylogenetic network, Rearrangement, Head-move operation, Tail-move operation, Network space, Diameter, 92D15, 68R10, 68R05, 05C20

## Abstract

Popular methods for exploring the space of rooted phylogenetic trees use rearrangement moves such as rooted Nearest Neighbour Interchange (rNNI) and rooted Subtree Prune and Regraft (rSPR). Recently, these moves were generalized to rooted phylogenetic networks, which are a more suitable representation of reticulate evolutionary histories, and it was shown that any two rooted phylogenetic networks of the same complexity are connected by a sequence of either rSPR or rNNI moves. Here, we show that this is possible using only *tail moves*, which are a restricted version of rSPR moves on networks that are more closely related to rSPR moves on trees. The connectedness still holds even when we restrict to distance-1 tail moves (a localized version of tail moves). Moreover, we give bounds on the number of (distance-1) tail moves necessary to turn one network into another, which in turn yield new bounds for rSPR, rNNI and SPR (i.e. the equivalent of rSPR on unrooted networks). The upper bounds are constructive, meaning that we can actually find a sequence with at most this length for any pair of networks. Finally, we show that finding a shortest sequence of tail or rSPR moves is NP-hard.

## Introduction

Leaf-labelled trees are routinely used in phylogenetics to depict the relatedness between entities such as species and genes. Accurate knowledge of these trees, commonly called *phylogenetic trees*, is vital for our understanding of the processes underlying molecular evolution, and thousands of phylogenetic trees are reconstructed from molecular data each day.

However, this representation is not suitable when reticulation events such as hybrid speciations (e.g. Abbott et al. 2013), horizontal gene transfers (e.g. Zhaxybayeva and Doolittle 2011) and recombinations (e.g. Vuilleumier and Bonhoeffer 2015) are involved in the evolution of the entities of interest. In such cases, a more suitable representation can be found in *phylogenetic networks*, where in its broadest sense a phylogenetic network can be thought of as a leaf-labelled graph (directed or undirected), usually without parallel edges and degree-2 nodes (Morrison 2011; Huson et al. 2010).

Common procedures used to reconstruct phylogenetic trees from biological data are tree rearrangement heuristics (Felsenstein 2004). These techniques consist of choosing an optimization criterion (e.g. maximum parsimony, maximum likelihood, a distance-based scoring scheme) or opting for a Bayesian approach, and then using *tree rearrangement moves* to explore the space of phylogenetic trees. These moves specify possible ways of generating alternative phylogenies from a given one, and their fundamental property is to be able to transform, by repeated application, any phylogenetic tree into any other phylogenetic tree. Several tree rearrangement moves have been defined in the past, the most commonly used ones being *Nearest Neighbour Interchange* (*NNI*) moves and *Subtree Prune and Regraft* (*SPR*) moves; when the phylogenetic trees are considered as *rooted*, i.e. directed and out-branching (i.e. singly rooted), we have their rooted versions: rNNI and rSPR moves.

**Fig. 1:**
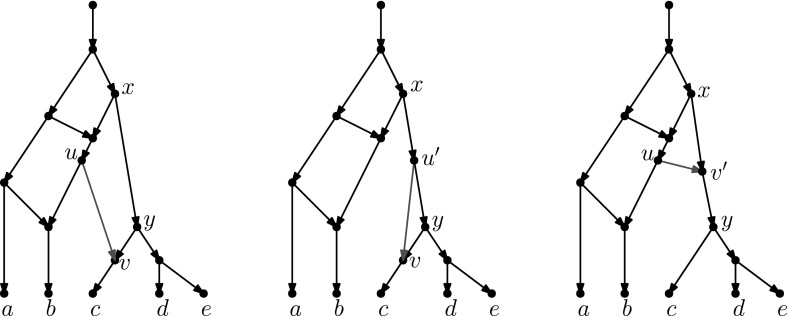
(Color figure online) Starting with the rooted phylogenetic network on the left, if we move the tail of (*u*, *v*) to (*x*, *y*) we produce the network in the middle. If we instead move the head of (*u*, *v*) to (*x*, *y*), we produce the network on the right

Recently, researchers have become interested in defining rearrangement moves for phylogenetic networks and studying their properties (Bordewich et al. 2017a; Francis et al. 2017; Gambette et al. 2017; Huber et al. 2016). Huber et al. (2016) gave a generalization of NNI moves for unrooted phylogenetic networks and showed the connectivity under these moves of the *tiers* of phylogenetic network space, i.e. phylogenetic networks having the same *reticulation number*. The latter concept will be formally defined in the next section, but roughly speaking, it is a way to express the amount of reticulate evolution present in a phylogenetic network. Francis et al. (2017) generalized SPR moves for unrooted phylogenetic networks and studied the properties of the NNI and SPR neighbourhoods of a network, giving bounds on their sizes. Gambette et al. (2017) focused on *rooted* phylogenetic networks, i.e. phylogenetic networks where the underlying graph is a rooted directed acyclic graph, and introduced generalizations of rNNI and rSPR moves for rooted phylogenetic networks. rSPR moves consist of *head moves* and *tail moves* (see Fig. 1), while rNNI moves can be seen as distance-1 head moves and distance-1 tail moves (see Definitions 2.5 and 2.6 for formal descriptions). In the same paper, Gambette et al. showed the connectivity of each tier of rooted phylogenetic networks under rNNI moves (and consequently also under rSPR moves) and gave bounds for the rNNI neighbourhood of a network. Finally, Bordewich et al. (2017a) introduced another generalization of rSPR called SNPR (SubNet Prune and Regraft), giving connectivity proofs and bounds for some classes of rooted phylogenetic networks, allowing parallel edges in most cases.

Note also that a handful of rearrangement heuristics for phylogenetic networks have been published recently (see, for example, PhyloNET (Than et al. 2008) and the BEAST 2 add-on SpeciesNetwork) and each of them uses its own set of rearrangement moves, e.g. the “Branch relocator” move in (Zhang et al. 2017) and the “Relocating the source of an edge” and “Relocating the destination of a reticulation edge” moves in (Yu et al. 2014). These sets of moves are often included among the ones cited in the previous paragraph; for example, the above-cited moves from (Yu et al. 2014) correspond, respectively, to head and tail moves (see Definitions 2.5 and 2.6) and their union correspond to the rSPR moves defined in (Gambette et al. 2017), c.f. next section. Since these papers do not focus on studying the properties of the moves they define, we will not discuss them here.

In this paper, we mostly focus on rooted binary phylogenetic networks and *tail moves*. In some sense, these can be seen as the most natural generalization of rSPR moves on rooted phylogenetic trees to networks. We show that each tier of rooted binary phylogenetic network space is connected using only tail moves, and even using only distance-1 tail moves. Hence, to get connectivity, head moves are not necessary. We note, however, that head moves could be useful in practice to escape from local optima (see the Discussion section). We also analyse the tail-move diameter, giving upper bounds on the number of tail moves, and the number of distance-1 tail moves ($\hbox {Tail}_1$ moves), necessary to turn any rooted binary phylogenetic network with *k* reticulations into any other such network on the same leaf set. Since the upper-bound proofs are constructive, we can actually find a sequence to go from one network to another network via tail moves. Interestingly, these bounds yield new bounds for rSPR, rNNI and SPR moves (see Table 1).

**Table 1:** New diameter bounds for several rearrangement moves

Move	Diameter of tier *k* networks with *n* leaves	
	Lower bound	Upper bound
rSPR	$n-\varTheta (\sqrt{n})$	$2n+3k$
Tail	$n-\varTheta (\sqrt{n})$	$3(n+2k)$
rNNI	$\varOmega (n \log (n))$	$O(n^2)$
$\hbox {Tail}_1$	$\varOmega (n \log (n))$	$O(n^2)$
SPR		$n+\frac{8}{3}k$

Finally, we show that the computation of a tail move sequence or rSPR sequence of shortest length is NP-hard.

## Definitions and Properties

### Phylogenetic Networks

In this subsection, we define the combinatorial objects of interest in this article.

#### Definition 2.1

A *rooted binary phylogenetic network* $N=(V,E)$ on a finite set *X* of taxa is a directed acyclic graph (DAG) with no parallel edges where the *leaves* (nodes of indegree-1 and outdegree-0) are bijectively labelled by *X*, there is a unique node of indegree-0 and outdegree-1—the *root*—and all other nodes are either *tree nodes* (indegree-1 and outdegree-2) or *reticulations* (indegree-2 and outdegree-1). We will write *V*(*N*), *L*(*N*) to denote the nodes and leaves of *N*, respectively. For a set of nodes $Y \subseteq V$, we will write *N*[*Y*] to denote the subgraph of *N* induced by *Y*, and we will write $N\setminus Y$ to denote $N[V \setminus Y]$. For any directed acyclic graph *D* (including rooted binary phylogenetic networks), we will write |*D*| to denote the number of nodes in *D*.

A *rooted binary phylogenetic tree* is a rooted binary phylogenetic network with no reticulation nodes (all edges are directed outward from the root). For brevity, we henceforth simply use the terms *network* and *tree*.

#### Definition 2.2

Let *N*, $N'$ be two directed acyclic graphs (including networks) with some nodes labelled with *X*. Then, an *isomorphism between* *N* *and* $N'$ is a bijection $\phi : V(N) \rightarrow V(N')$ such that

- Two nodes $u,v \in V(N)$ are adjacent in *N* if and only if $\phi (u)$ and $\phi (v)$ are adjacent in $N'$;
- For any labelled node $u \in L(N)$, $\phi (u)$ is the node in $L(N')$ that has the same label as *u*.

We say *N* and $N'$ are *isomorphic* if there exists an isomorphism between *N* and $N'$.

Let *u* and *v* be nodes in a network, and then we say *u* is *above* *v* and *v* is *below* *u* if $u \le v$ in the order induced by the directed graph underlying the network. Similarly, if $e=(x,y)$ is a directed edge of the network and *u* a node, then we say that *e* is *above* *u* if *y* is above *u* and *e* is *below* *u* if *x* is below *u*. Let $e=(u,v)$ be an edge of a phylogenetic network, then we say that *u* is the *tail* of *e* and *v* is the *head* of *e*. In this situation, we also say that *u* is a *parent* of *v*, or *u* is *directly above* *v* and *v* is a *child* of *u* or *v* is *directly below* *u*. Note that in a tree *T*, there is always a unique *lowest common ancestor* (LCA) per pair of nodes of *T*. This is not the case for networks, where we can have several different LCAs.

A standard measure of tree-likeness of a network *N* is the *reticulation number*. Denoted by *r*(*N*), it is defined as the (unique) number of edges that need to be removed in order to obtain a tree. As any tree has exactly one more node than edges, we may equivalently define it as:

$$\begin{aligned} r(N)=\sum _{v\in V : \delta ^{-}(v)>0 }\delta ^{-}(v)-1 =|E| - |V|+1, \end{aligned}$$

where $\delta ^{-}(v)$ denotes the indegree of the node *v*. Note that for binary networks, *r*(*N*) is equal to the number of reticulation nodes.

Here, we are interested in studying the sets of networks with the same reticulation number:

#### Definition 2.3

Let *X* be a finite set of taxa. The *kth tier* (Francis et al. 2017; Huber et al. 2016) on *X* is the set of all networks with label set *X* and reticulation number *k*.

This interest comes from the fact that comparing optimization scores across networks with different reticulation numbers may be tricky, since a network generally allows a better fit with the data when its reticulation number is higher. For this reason, it is increasingly conventional (e.g. in Gambette et al. 2017) to make a distinction between “horizontal” rearrangement moves, enabling the exploration of a tier, and “vertical” moves, allowing a jump across tiers.

The next observation will be useful in the next sections:

#### Observation 2.4

As every non-root, non-leaf node in a binary network is incident to exactly three edges, we have that $3|V| = 2|E| + 2|X| + 2$. Subtracting $2|V| = 2|E| - 2k + 2$ from each side, we get that $|V| = 2|X| + 2k$, which in turn implies that $|E| = 2|X| + 3k - 1$. Thus, any two binary networks in the *k*th tier on *X* have the same number of nodes and edges, not just the same number of reticulation nodes.

### Rearrangement Moves

As mentioned in the introduction, several analogues of rearrangement moves on trees have been defined for phylogenetic networks. Such rearrangement moves typically modify the head, the tail, or both the head and the tail of one edge. These moves are only allowed if they produce a valid phylogenetic network.

**Fig. 2:**
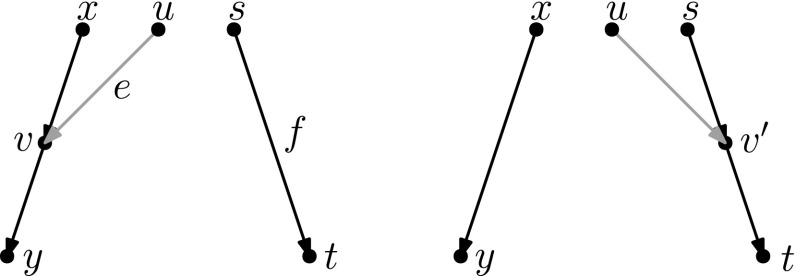
(Color figure online) A head move as described in Definition 2.5

#### Definition 2.5

(*Head move*) Let $e=(u,v)$ and *f* be edges of a network. A head move of *e* to *f* consists of the following steps (Fig. 2):

1. Deleting *e*;
2. *Subdividing* *f* with a new node $v'$;
3. *Suppressing* the indegree-1 outdegree-1 node *v*;
4. Adding the edge $(u,v')$.

*Subdividing* an edge (*u*, *v*) consists of deleting it and adding a node *x* and edges (*u*, *x*) and (*x*, *v*). *Suppressing* an indegree-1, outdegree-1 node *x* with parent *u* and child *v* consists in removing edges (*u*, *x*) and (*x*, *v*) and node *x*, and then adding an edge (*u*, *v*).

Head moves are only allowed if the resulting digraph is still a network, see Definition 2.1. We say that a head move is a *distance-d* move if, after step 2, a shortest path from *v* to $v'$ in the underlying undirected graph has length $d+1$ (number of edges in the path).

#### Definition 2.6

**Fig. 3:**
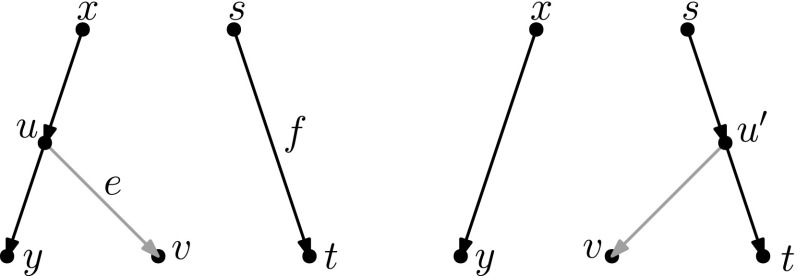
(Color figure online) A tail move as described in Definition 2.6

(*Tail move*) Let $e=(u,v)$ and *f* be edges of a network. A tail move of *e* to *f* consists of the following steps (Fig. 3):

1. Deleting *e*;
2. Subdividing *f* with a new node $u'$;
3. Suppressing the indegree-1 outdegree-1 node *u*;
4. Adding the edge $(u',v)$.

Tail moves are only allowed if the resulting digraph is still a network, see Definition 2.1. We say that a tail move is a *distance-d* move if, after step 2, a shortest path from *u* to $u'$ in the underlying undirected graph has length $d+1$.

Note that head moves are only possible for *reticulation edges*, that is, edges in which the head is a reticulation. This means that these moves are, in our opinion, not necessarily part of a natural generalization of rSPR moves on trees, which consist only of tail moves.

Other generalizations of tree moves that have been proposed include head moves. For example, one *rSPR move* (Gambette et al. 2017) on a network consists of one head move or one tail move, and one *rNNI move* consists of one distance-1 head move or one distance-1 tail move. Thus, it is clear that any tail move is an rSPR move, and any distance-1 tail move is an rNNI move. *SNPR moves* (Bordewich et al. 2017a) are a variation on the theme: they are defined on networks where parallel edges are allowed, as a tail move or a deletion/addition of an edge. Because the deletion/addition of an edge is a vertical move, SNPR moves can change the reticulation number. Moreover, even if vertical moves are not permitted, the presence of parallel edges makes this restriction of SNPR moves still subtly different from the tail moves studied in this paper, see Definition 2.6. Nevertheless, Bordewich et al. do disallow parallel edges and vertical moves when studying *tree-child networks* (i.e. networks in which every internal node has at least a child that is not a reticulation) and they prove that any tier of tree-child networks is connected by tail moves if $k\le |X|-2$.

**Fig. 4:**
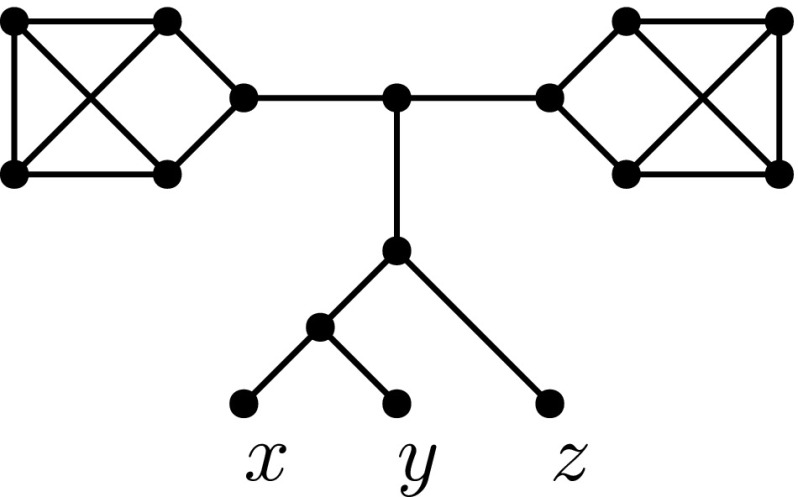
An unrootable unrooted network

Rearrangement moves are also defined for *unrooted networks*: connected graphs with nodes of degree 1 (the leaves) and of degree 3, where the leaves are labelled bijectively with some set *X*, with $|X|\ge 2$. The unrooted equivalents of rSPR moves and rNNI moves are called *SPR moves* and *NNI moves*, respectively (Huber et al. 2016). An SPR move relocates one of the endpoints of an edge, like an rSPR move, with the condition that the resulting graph is still an unrooted network. An NNI move on an unrooted network is again the distance-1 version of an SPR move. Moreover, any rSPR move induces an SPR move on the underlying undirected graph, and similarly for rNNI and NNI moves. This means that, in some sense, any rSPR move is an SPR move, and any rNNI move is an NNI move. The converse is clearly not true: for example, an rSPR move that creates a directed cycle is invalid, but the induced SPR move on the undirected network may be valid. In addition, not every unrooted network is the underlying graph of a rooted network (see for example the network in Fig. 4). Such networks are unrootable and will be treated in more detail in Sect. 4.4.

### Properties of Tail Moves

From now on, we will mostly focus on tail moves (and hence on rooted networks). Note that not all edges of a network can be moved by a tail move; we introduce here the notion of *movability* of edges:

#### Definition 2.7

Let $e=(u,v)$ be an edge in a network. Then, *e* is called *non-movable* if *u* is the root, if *u* is a reticulation, or if the removal of *e* followed by suppressing *u* creates parallel edges. Otherwise, *e* is called *movable*.

There is only one situation in which moving a tail of an edge (*u*, *v*) can result in parallel edges: when there exists an edge from the parent of *u* to the child of *u* other than *v*. The situation is characterized in the following definition:

#### Definition 2.8

Let *N* be a network and let *x*, *u*, *y* be nodes of *N*. We say *x*, *u* and *y* form a *triangle* if there are edges (*x*, *u*), (*u*, *y*) and (*x*, *y*). The edge (*x*, *y*) is called the *long edge* and (*u*, *y*) is called the *bottom edge* of the triangle.

The interesting case in Definition 2.8 is when *u* is a tree node:

#### Observation 2.9

Let *x*, *u* and *y* form a triangle in a network, and let *v* be the other child of *u*. The edge (*u*, *v*) is non-movable because this move would create parallel edges from *x* to *y*. The edges (*u*, *y*) and (*x*, *y*) are movable, however, and if (*u*, *y*) is moved sufficiently far up (i.e., destroying the triangle) then the new edge (*x*, *v*) is also movable.

The following observation is a direct consequence of Observation 2.9 and of the binary nature of the networks studied in this paper, and will play an important role in the arguments presented in the next section:

#### Observation 2.10

Let *u* be a tree node, then at least one of its child edges is movable because at most one of these has its tail in a triangle not containing its head.

Note that movability of an edge *e* does not imply that there exists a valid tail move for *e*: it only ensures that we can remove the tail without creating a clear violation of the definition of a network; it does not ensure that we can reattach it anywhere else. The following observation characterizes valid moves:

#### Observation 2.11

The tail of an edge $e=(u,v)$ can be moved to another edge $f=(s,t)$ if and only if the following conditions hold:

1. *e* is movable;
2. *f* is not below *v*;
3. $$t\ne v$$.

The first condition assures that the tail can be removed, the second that we do not create cycles, and the third that we do not create parallel edges. Note that these conditions imply that *moving* a (movable) edge *up* is allowed, i.e. moving an edge to another edge that is above it. In particular, a tail can always be moved to the root edge. However, note that it is not certain that this results in a non-isomorphic network.

The following lemma is related to the previous observation and will be used in the next section to find a tail that can be moved down “sufficiently far” (see Cases (b) and (d) in the proof of Lemma 3.3):

#### Lemma 2.12

Let *x*, *y* be nodes of a phylogenetic network *N* such that neither *x* nor *y* is an LCA of *x* and *y*. Then, for any LCA of *x* and *y*, one of the child edges of that LCA is a movable edge that is not both above *x* and above *y*.

#### Proof

Consider an arbitrary LCA *u* of *x* and *y*. This LCA is a tree node, and both child edges are above either *x* or *y*, but not both. Because at least one of the child edges of a tree node is movable (by Observation 2.10), at least one of the child edges of the LCA has the desired properties. $\square$

We conclude this section by citing a result by Gambette et al. that implies connectivity of *k*th tiers (for any $k\ge 0$) via *rNNI moves*, which are equivalent to the combination of distance-1 head moves and distance-1 tail moves. This result is fundamental for our proof of connectivity of the tiers via tail moves.

#### Theorem 2.13

(Theorem 3.2 in Gambette et al. 2017) Let *N* and $N'$ be two rooted binary phylogenetic networks belonging to the *k*th tier on a fixed leaf set *X*. Then, there exists a sequence of rNNI moves turning *N* into $N'$.

## Head Moves Rewritten

**Fig. 5:**
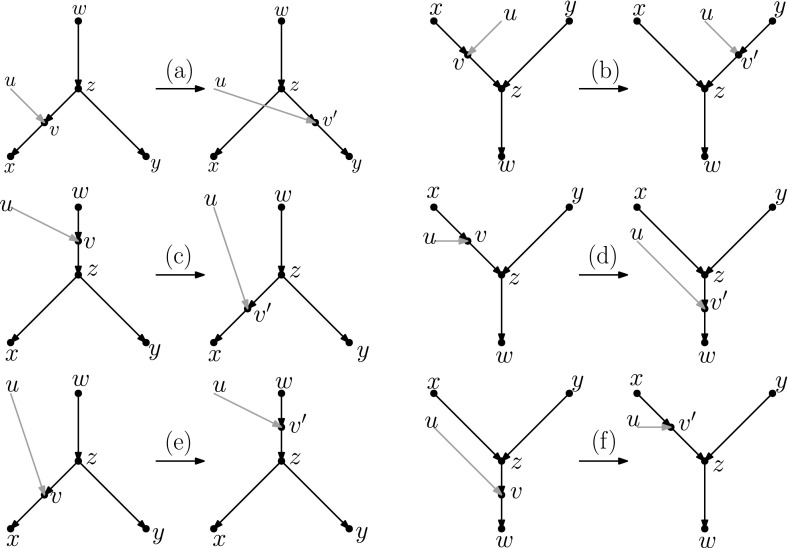
(Color figure online) Illustration of all possible distance-1 head moves: **a** sideways movement below a tree node, **b** sideways movement above a reticulation, **c** downward movement through a tree node, **d** downward movement through a reticulation, **e** upward movement through a tree node, **f** upward movement through a reticulation

In this section, we present one of the main results of the paper: the connectivity of *k*th tiers using tail moves. Theorem 2.13 tells us that *k*th tiers are connected by rNNI moves. This means that, to prove our result, it suffices to show that any distance-1 head move can be replaced by a sequence of tail moves. To this end, we list in Fig. 5 all different cases where it is possible to perform a distance-1 head move. We observe the following:

### Observation 3.1

Each distance-1 head move is depicted as exactly one of the cases (a)–(f) in Fig. 5. Note that the figure does not indicate whether *u*, *w*, *x*, *y* are all distinct. There are only a few cases in which *u*, *w*, *x*, *y* are not all distinct, and the head move is valid. If $x=y$ in cases (a) and (b) or $u=y$ in cases (d) and (f), we have a valid move that results in a network that is isomorphic to the starting one. Having $u=w$ in case (a) is the only situation that results in a non-isomorphic network. All other possibilities lead to invalid moves.

### Observation 3.2

It is easy to see that moves (c) and (e) as well as moves (d) and (f) are each others reversions. Because all tail moves are also reversible, we only have to show that moves (a)–(d) can be rewritten as a sequence of tail moves.

We treat all cases separately in the following lemma. We note here that, in the proof of this lemma, the sequences of tail moves used to mimic the different distance-1 head moves are often non-unique and possibly non-optimal. In the following, we shall use the convention of naming $t'$ the new tail node created when moving an edge with tail *t*.

### Lemma 3.3

All distance-1 head moves can be substituted by a sequence of tail moves, except for the head move in the network depicted on the right in Fig. 8. For networks with more than one leaf, a sequence of length at most four can be found.

### Proof

We analyse Cases (a)–(d) separately.
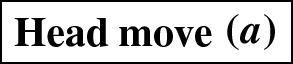
 In light of Observation 3.1, we distinguish two cases: $u\ne w$ and $u=w$. All other nodes in Fig. 5a are distinct.

**Fig. 6:**
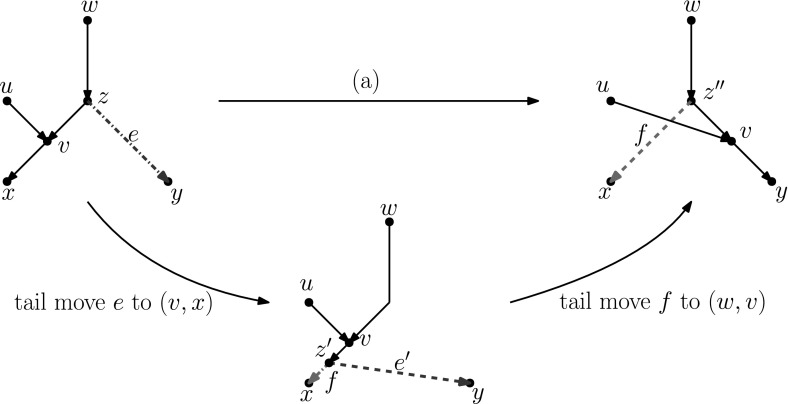
(Color figure online) Proof of Lemma 3.3: the sequence of tail moves needed to simulate head move (a) in Case 1. Moving edges are dash-dotted before a move, and dashed after a move

**Fig. 7:**
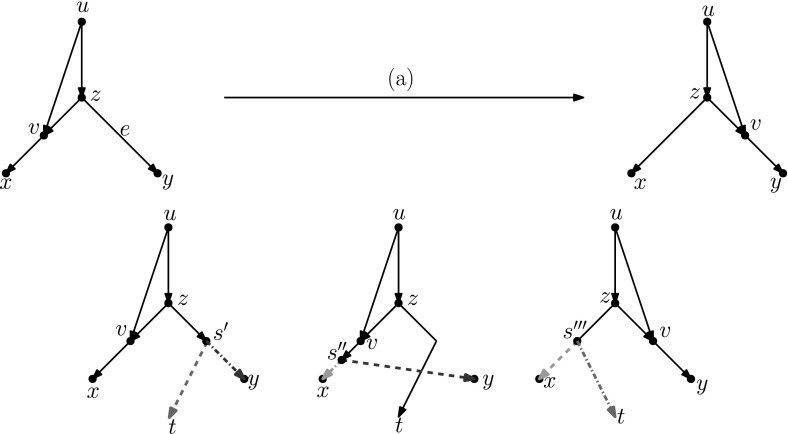
(Color figure online) Proof of Lemma 3.3: the sequence of tail moves used to simulate head move (a) in Case 2. The “extra tail” *s* of edge (*s*, *t*) is used in the sequence of moves: (*s*, *t*) to (*z*, *y*), $(s',y)$ to (*v*, *x*), $(s'',x)$ to (*z*, *t*) and $(s''',t)$ to (*p*, *r*). Here, *p* and *r* are, respectively, the parent and (other) child of *s* in the network before the head move

**Fig. 8:**
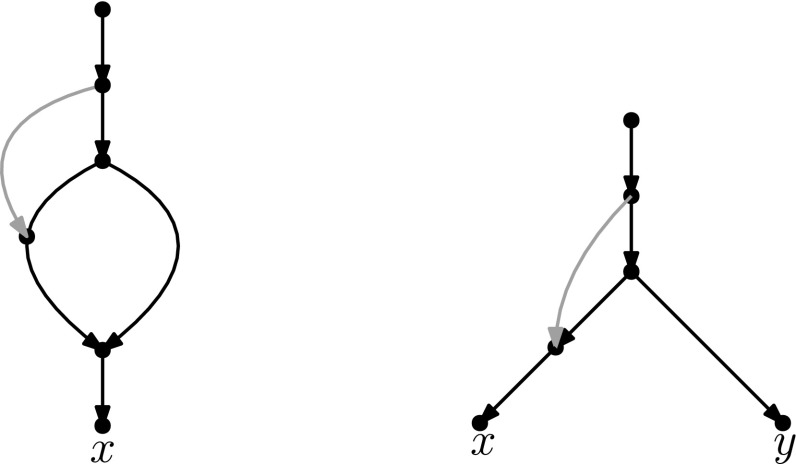
(Color figure online) The networks for which there are no valid tail moves to simulate a distance-1 head move of type (a). Left: The network with one leaf and 2 reticulations where all valid head moves give isomorphic networks. Right: A network in which one non-trivial head move of type (a) is possible. There are no non-trivial tail moves for this network, therefore the head move cannot be simulated by tail moves

1. $\varvec{u\ne w}$ In this case, we can use the sequence of tail moves depicted in Fig. 6, since the validity of the head move implies that the intermediate network does not contain directed cycles and parallel edges, as we shall show in the following.
  To see that the intermediate network has no directed cycles or parallel edges, we check that the tail move *e* to (*v*, *x*) is valid. Note that *e* is movable (Definition 2.7) because *z* is not the root nor a reticulation, and because $u\ne w$, the removal of the tail of (*z*, *y*) does not create parallel edges. Moreover, by Observation 2.11, *e* can be moved to (*v*, *x*) since (*v*, *x*) is not below *y* (otherwise there would be a path from *y* to *v* in the network after the head move, which implies a directed cycle, and the head move could not be valid) and all nodes in Fig. 5 are distinct, so in particular $x\ne y$.
  Thus, we can conclude that the intermediate network is valid, and therefore that the sequence of tail moves is valid.
2. $\varvec{u=w}$ Note that *u* and *v* form a triangle together with the tree node *z*. In this situation, we cannot directly use the same sequence as before, since this sequence would create parallel edges. There are two conditions under which we can solve this problem:
  (i) **There is a tree node somewhere not above** $\varvec{u}$, **that is not** *u* or *z*. In this case, we “expand” the triangle. Instead of moving edge *e* directly, we first subdivide it by moving a tail to *e*. Then, we can apply the sequence of moves depicted in Fig. 7. Barring the addition of the “extra tail”, the sequence of moves is quite similar to the moves in Case 1, and it can be shown in a similar way that this will not create cycles or parallel edges.
  (ii) **There is at least one vertex above** $\varvec{u}$ **in addition to the root** In this case we “destroy” the triangle. The bottom edge of the triangle (*z*, *v*) is movable. If this edge is moved to the root (i.e. to the single edge incident to the root), the situation changes to that of Case 1. Thus, we can apply the sequence moves for that case, and move the bottom edge of the triangle back. Since (*z*, *v*) is movable, moving up and down to/from the root are valid moves by Observation 2.11.
  If neither of these conditions hold, then there are no tree nodes except *u* and *z*. It follows by inspection that one of two possibilities holds - either *x* and *y* are the same reticulation node with a single leaf below it, or *x* and *y* are both leaves. The two possible networks are shown in Fig. 8. In the first network, no head move leads to a different (non-isomorphic) network. The only non-trivial case is the second network: here the type (a) head move cannot be substituted by a sequence of tail moves, because there is no valid tail move. Note that this network is excluded in the statement of the lemma.

**Fig. 9:**
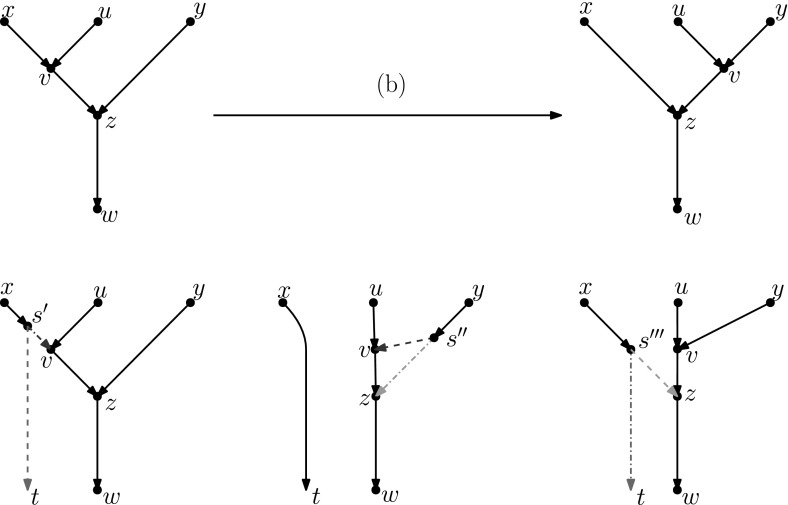
(Color figure online) Proof of Lemma 3.3: the sequence of tail moves used to simulate head move (b). The moving edges are coloured, and the colouring of the edges is consistent throughout the sequence. The tail of the extra edge (light blue) is first moved to (*x*, *v*), then the orange edge $(s',v)$ is moved to (*y*, *z*), then the green edge $(s'',z)$ is moved to (*x*, *t*), and finally the light blue edge $(s''',t)$ (the “extra tail”) is moved back


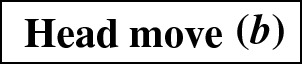
 The idea of this substitution is to use an extra tail again. The sequence we use is given in Fig. 9. The main challenge is to find a usable tail: to use the sequence of moves, we need a tail that can be moved below either *x* or *y*. We will find this tail by considering an LCA *s* of *x* and *y* in Fig. 9. We treat the following cases:

1. **The LCA** $\varvec{s}$ **is neither equal to** $\varvec{x}$ **nor to** $\varvec{y}$ At least one of the child edges (*s*, *t*) of this LCA can be moved and it is not above both *x* and *y* (by Lemma 2.12). Hence, we can move this tail down below one of these nodes (by Observation 2.11).
  (i) **The movable edge** $\varvec{(s,t)}$ **is above** $\varvec{y}$ If *t* is not directly below *x*, we can use the proposed sequence of moves. However, if *t* is directly below *x*, then *x* is above *y*, and *x* is the only LCA of *x* and *y*. This contradicts our assumption that $s\ne x$ and we can certainly use the sequence of moves depicted in Fig. 9.
  (ii) **The movable edge** $\varvec{(s,t)}$ **is above** $\varvec{x}$ The symmetry of the situation and the reversibility of the sequence of moves lets us reduce to the previous case: We do the sequence of moves in reverse order, where we switch the labels of *x* and *y*.
2. $\varvec{x}$ **is an LCA of** $\varvec{x}$ **and** $\varvec{y}$ Because *x* is the LCA of *x* and *y* and there is no path from *v* to *y* (such a path would imply a path from *z* to *y* and thus a cycle in the starting network), *x* must be a tree node. Let the children of *x* be *v* and *t*, and let the parent of *x* be *p*. Two cases are possible:
  (i) $\varvec{(x,v)}$ **is movable** In this case, we can use a similar sequence as before, but without the addition of a tail: (*x*, *v*) to (*y*, *z*), $(x',z)$ to (*p*, *t*).
  (ii) $\varvec{(x,v)}$ **is not movable** In this case, *p*, *x* and *t* form a triangle. We employ a strategy to break the triangle similar to the one used for Case 2 of head move (a).
    **There is a node above the triangle besides the root** Moving the long edge of the triangle to the root, we can reduce the problem to Case 2i. Then, we apply the same sequence of tail moves and move the long edge back to the original position.
    **The node above the triangle is the root** If there is no node above the triangle, then there is a tree node not above *x*, for example an ${{\mathrm{LCA}}}$ *s* of *u* and *y*. It is easy to see that *s* cannot be above *x* since the parent node of *x* has children *x* and *t* and its parent is the root. Note that *s* can only be a reticulation if it has outdegreee 1, i.e. if $s=u$ or $s=y$. Suppose that $s=u$. Since *v* and *z* are reticulations, this would mean that *y* is below *z*, which implies the existence of a directed cycle in the original network, a clear contradiction. A similar reasoning shows that $s=y$ leads to a contradiction. Hence, *s* is a tree node and one of the child edges can be moved up to (*x*, *t*). This puts us in the situation of Case 2i, and we can use the corresponding sequence of moves. After the corresponding sequence of moves, we move the edge back to its original position.
3. $\varvec{y}$ **is an LCA of** $\varvec{x}$ **and** $\varvec{y}$ Like in Case 1ii, we use the symmetry of the situation and the reversibility of the moves to reduce to the previous case (Case 2) by doing the moves in reverse order where the labels of *x* and *y* are switched.

**Fig. 10:**
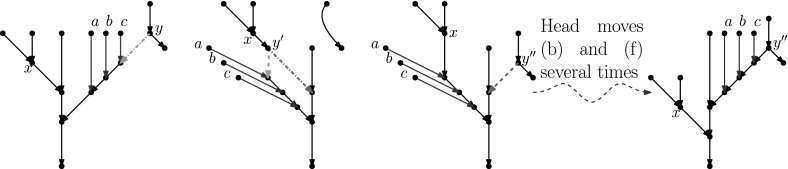
(Color figure online) Proof of Lemma 3.3: the sequence of tail moves used to simulate head move (d) in Case 3iii. All reticulations on the path to the nearest tree node above it move with it to the other side

In all sequences we gave, no directed cycles were created, because such cycles imply directed cycles in one of the networks before and after the head move. We conclude that any allowed head move of type (b) can be substituted by a sequence of tail moves.
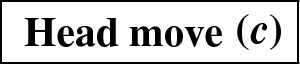
 This is the easiest case, as we can substitute the head move by exactly one tail move: (*z*, *y*) to (*w*, *v*), with labelling as in Fig. 5. Parallel edges and directed cycles cannot occur because there are no intermediate networks.
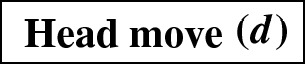
 This one is the most complicated distance-1 head move to translate in tail moves. We exploit the following symmetry of this case: relabelling $u\leftrightarrow y$ and $v\leftrightarrow z$ transforms the network before the head move into the network after the head move. Let us start with the easiest case:

1. **Either** $\varvec{(u,v)}$ **or** $\varvec{(y,z)}$ **is movable** First assume that (*u*, *v*) is movable and that the other child and parent of *u* are *p* and *t*, we move (*u*, *v*) to (*y*, *z*) and then $(u',z)$ to (*p*, *t*). The case that (*y*, *z*) is movable can be tackled in a similar way thanks to the reversibility of tail moves and the symmetry given above.
2. **Either** $\varvec{u}$ **or** $\varvec{y}$ **is a tree node** If one of them is movable, we are in the previous situation, otherwise we mimic the approach of Case 2ii of head move (b). Assume without loss of generality that *y* is at the side of a triangle. We either move the bottom edge of the triangle up to the root, or we use a child edge of a tree node to subdivide the bottom edge of the triangle. In the latter case, we can take, for example, ${{\mathrm{LCA}}}(x,u)$, which is a tree node because *x* and *u* are both directly above *v*, and which is not above *y*.
3. **Both** $\varvec{u}$ **and** $\varvec{y}$ **are reticulations** In this case, we try to recreate the situation of Case 1 by adding a tail on one of the edges (*u*, *v*) and (*y*, *z*).
  (i) **The network contains at least two leaves** We can pick two distinct leaves, at least one of which is below *w*. Suppose first that both of these leaves are below *w*, then an LCA of the leaves is also below the reticulation and one of its child edges can be used as the extra tail by Lemma 2.12. If only one of the leaves is below the reticulation, then any LCA of the leaves has one edge that is not above *w*. If this edge can be moved, we can directly use it; otherwise, we first move the lower part of the triangle to the root, and then still use this edge.
  (ii) **The network contains one leaf, and** $\varvec{w}$ **is not this leaf** The only leaf is below the lowest reticulation *r* of the network, which is not *z*. Let the parents of *r* be $p_1$ and $p_2$. To find a usable extra tail, we use the same argument as in Case 3i but now with $p_1$ and $p_2$ instead of the two leaves. Note that ${{\mathrm{LCA}}}(p_1,p_2)$ is a tree node because both $p_1$ and $p_2$ are directly above *r*.
  (iii) **The only leaf in the network is** $\varvec{w}$ We have not found a way to solve this case ‘locally’ as we did before. Go up from these reticulations to some nearest tree node. The idea, then, is to use one tree edge to do the switch as in the case we discussed previously (Fig. 10).
    The result is that all reticulations on the path to the nearest tree node move with the tree edge. These reticulations can be moved back to the other side using the previously discussed moves. In particular, we move the heads sideways using head move (b), and then we move them up using head move (f) where the main reticulation (*z*) is not the lowest reticulation in the network.

$$\square $$

Using Theorem 2.13 and the above lemma, we directly get the connectivity result for tail moves.

### Theorem 3.4

Let *N* and $N'$ be two networks in the *k*th tier on *X*. Then there exists a sequence of tail moves turning *N* into $N'$, except if $k=1$ and $|X|=2$.

## Distances and Diameter Bounds

Given a class of moves $\mathcal {M}$ and two networks $N,N'$, define the $\mathcal {M}$*-distance* $d_{\mathcal {M}}(N,N')$ to be the minimum length of a sequence of moves in $\mathcal {M}$ turning *N* into $N'$, or $\infty$ if no such sequence exists. Also, denote by $\varDelta _k^{\mathcal {M}}(n)$ the *diameter* of the *k*th tier with *n* leaves with respect to $\mathcal {M}$; that is, the maximum value of $d_{\mathcal {M}}(N,N')$ over any pair of networks $N,N'$ belonging to the *k*th tier on a fixed leaf set *X* with $|X|=n$. In this section, we mostly focus on the tail distance, studying its diameter and the complexity of computing such a distance between two networks. Interestingly enough, the findings on the tail distance will also yield results for the rNNI, rSPR and SPR distances. Indeed, tail moves are related to other types of moves such as rNNI and rSPR, which has implications for their induced metrics and diameters. There are obvious bounds when a class of moves contains another class of moves, by which we mean that each move of the second class is equivalent to a move of the first class (e.g. $\text {rNNI}\subseteq \text {rSPR}$). Then, we have that $\mathcal {M}\subseteq \mathcal {M}'$ implies $\varDelta _k^{\mathcal {M}}(n)\ge \varDelta _k^{\mathcal {M}'}(n)$. This observation will be very useful in the rest of this section.

### The Complexity of Computing the Tail Distance and the rSPR Distance

In this subsection, we prove that computation of the tail and rSPR distance between two networks is NP-hard:

#### Theorem 4.1

Computing the rSPR distance and the tail distance between two networks is NP-hard for any tier of phylogenetic network space.

To prove the theorem, we shall introduce several new concepts.

#### Definition 4.2

A *mycorrhizal forest*^1^ with leaves *X* is a network $M=(\mathcal {T},R)$ defined by:

- A set of *t* trees $\mathcal {T} = (T_1,\ldots ,T_t)$ with leaf sets $X_1,\ldots ,X_t$, respectively (the *tree components*),
- A rooted binary phylogenetic network *R* (the *root component*) with *t* leaves $x_1,\ldots ,x_t$, such that deleting all these leaves and the root makes *R* biconnected.

**Fig. 11:**
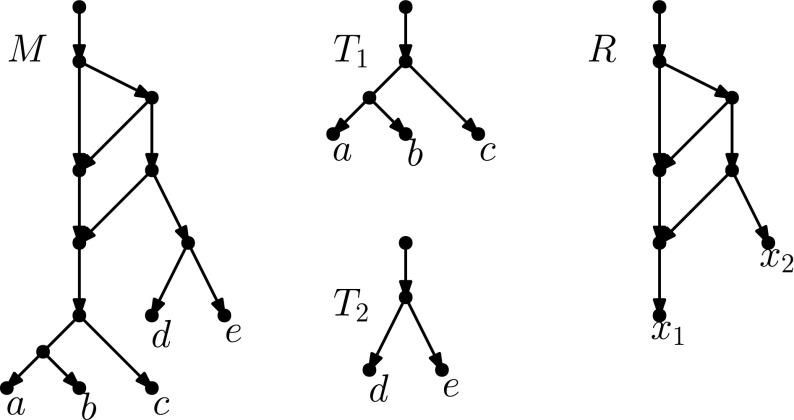
From left to right: a mycorrhizal forest $M=(\mathcal {T},R)$, its tree components $\mathcal {T} = (T_1,T_2)$ and the root component *R*

The mycorrhizal forest is the network where the root edge of $T_i$ is identified with the edge leading to leaf $x_i$ in *R* (see Fig. 11 for an example). A mycorrhizal forest with $t=1$ is called a *mycorrhizal tree*.

If *M* and $M'$ are mycorrhizal forests with tree components $T_1,\ldots ,T_t$ and $T'_1,\ldots ,T'_t$, respectively, both having leaf sets $X_1,\ldots ,X_t$, then we denote $d_{\text {treeSPR}}(M,M'):=\sum _{i}d_{\text {rSPR}}(T_i,T_i')$, which is the distance induced by rSPR moves only within tree components (treeSPR moves).

#### Definition 4.3

Let *T* be a tree on *X* and let $Y\subseteq X\cup \{\rho \}$, where $\rho$ denotes the root of *T*. Then, $T|_Y$ is the subtree of *T* induced by *Y*: Take the union of all shortest paths between nodes of *Y*, and then suppress all indegree-1 outdegree-1 vertices.

#### Definition 4.4

Let $\mathcal {T}=\{T_i\}_{1\le i\le t}$ be a set of trees with labels *X*. An *agreement forest* (AF) of $\mathcal {T}$ is a partition $\{X_j\}$ of $X\cup \{\rho \}$ (where $\rho$ denotes the root), such that all $T_i|_{X_j}$ are isomorphic for each fixed *j* and all $T_i|_{X_j}$ are node-disjoint for a fixed *i*.

#### Lemma 4.5

Let *M* and $M'$ be two mycorrhizal forests with the same root component and tree sets $\{T_i\}_{1\le i\le n}$ and $\{T'_i\}_{1\le i\le n}$ with leaf sets $X_i=X_i'$. Then,

$$\begin{aligned} d_\mathrm{tail}(M,M')=d_\mathrm{rSPR}(M,M')=d_\mathrm{treeSPR}(M,M'). \end{aligned}$$

#### Proof

Clearly, $d_\mathrm{rSPR}\le d_\mathrm{treeSPR}$ and $d_\mathrm{tail}\le d_\mathrm{treeSPR}$, because a treeSPR sequence is also a tail sequence and a rSPR sequence.

Now suppose we have a sequence of tail moves or rSPR moves from *M* to $M'$. Because both networks have all reticulations in the root component, deleting all moving edges gives agreement forests on the trees $T_i$ and $T_i'$ for each *i*. These agreement forests each have size larger than or equal to the corresponding maximum agreement forest (MAF). Because the rSPR distance is equal to the size of a MAF (Bordewich and Semple 2005) minus one, we conclude that the number of moves in the rSPR or tail sequence is larger than or equal to the needed number of moves in all treeSPR sequences between $T_i$ and $T_i'$ together. Hence, we conclude $d_\mathrm{rSPR}\ge d_\mathrm{treeSPR}$ and $d_\mathrm{tail}\ge d_\mathrm{treeSPR}$. $\square$

Theorem 4.1 follows directly from this lemma, because computing treeSPR distance is NP-hard (Bordewich and Semple 2005). Indeed, for any *k* we can let *R* be a network with *k* reticulations that becomes biconnected after deleting the root and all leaves. Then calculating the rSPR or tail distance between $M = (\mathcal {T},R)$ and $M' = (\mathcal {T}',R)$ is equivalent to calculating the rSPR distance between $T_i$ and $T_i'$ for each *i*, and *M* and $M'$ are in the *k*th tier.

### The Diameter of Tail and rSPR Moves

In this subsection, we study the diameter bounds of tail move operations, i.e. the maximum value of $d_\mathrm{Tail}(N,N')$ over all possible networks *N* and $N'$ with the same reticulation number. In the following, we shall use the convention of naming $t_1$ the new tail node created when moving an edge with tail *t*, and naming $t_{i+1}$ the node created when moving an edge with tail $t_i$.

Given a network *N* and a set of nodes *Y* in *N*, we say *Y* is *downward-closed* if for any $u \in Y$, every child of *u* is in *Y*.

#### Lemma 4.6

Let *N* and $N'$ be networks in the *k*th tier on *X* such that *N* and $N'$ are not the networks depicted in Fig. 8. Let $Y \subseteq V(N), Y' \subseteq V(N')$ be downward-closed sets of nodes such that $L(N)\subseteq Y, L(N') \subseteq Y'$, and *N*[*Y*] is isomorphic to ${N'[Y']}$. Then there is a sequence of at most $3|N\setminus Y|$ tail moves turning *N* into $N'$.

#### Proof

We first observe that any isomorphism between *N*[*Y*] and ${N'[Y']}$ maps reticulations (tree nodes) of *N* to reticulations (tree nodes) of $N'$. Indeed, every node in *N*[*Y*] is mapped to a node in ${N'[Y']}$ of the same outdegree, and the tree nodes are exactly those with outdegree 2. It follows that *Y* and $Y'$ contain the same number of reticulations and the same number of tree nodes. As *N* and $N'$ have the same number of nodes and reticulations by Observation 2.4, it also follows that $V(N)\setminus Y$ and $V(N')\setminus Y'$ contain the same number of reticulations and the same number of tree nodes.

We prove the claim by induction on $|N \setminus Y|$. If $|N \setminus Y| = 0$, then $N = N[Y]$, which is isomorphic to ${N'[Y']} = N'$, and so there is a sequence of 0 moves turning *N* into $N'$.

If $|N \setminus Y| = 1$, then as *Y* is downward-closed, $N \setminus Y$ consists of $\rho _N$, the root of *N*, and by a similar argument $N '\setminus Y'$ consists of $\rho _{N'}$. Let *x* be the only child of $\rho _N$, and note that in *N*[*Y*], *x* is the only node of indegree 0, outdegree 2. It follows that in the isomorphism between *N*[*Y*] and ${N'[Y']}$, *x* is mapped to the only node in ${N'[Y']}$ of indegree 0, outdegree 2, and this node is necessarily the child of $\rho _{N'}$. Thus, we can extend the isomorphism between *N*[*Y*] and ${N'[Y']}$ to an isomorphism between *N* and $N'$ by letting $\rho _N$ be mapped to $\rho _{N'}$. Thus, again there is a sequence of 0 moves turning *N* into $N'$.

**Fig. 12:**
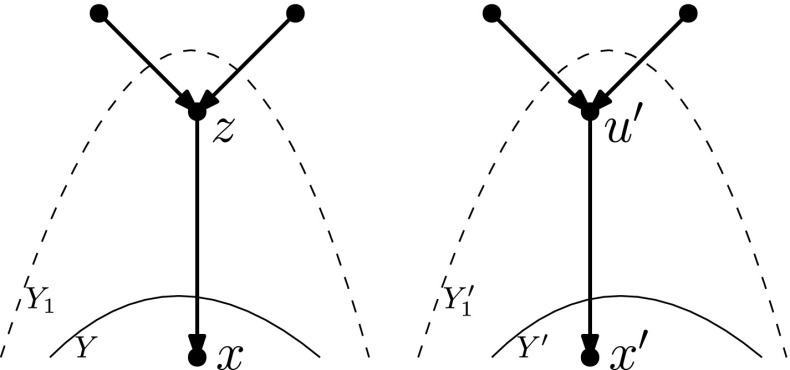
Proof of Lemma 4.6, Case 1a: If $u'$ is a lowest reticulation in $N' \setminus Y'$ with child $x'$, and the node $x \in Y$ corresponding to $x'$ has a reticulation parent *z* in $N \setminus Y$, then we may add *z* to *Y* and $u'$ to $Y'$

So now assume that $|N \setminus Y| > 1$. We consider three cases, which split into further subcases. In what follows, a *lowest node of* $N \setminus Y$ (*or* $N' \setminus Y'$) is a node *u* in $V(N)\setminus Y$ (or $V(N')\setminus Y'$) such that all descendants of *u* are in *Y* ($Y'$). Note that such a node always exists, as *Y* ($Y'$) is downward-closed and $L(N)\subseteq Y$ ($L(N') \subseteq Y'$).

**Fig. 14:**
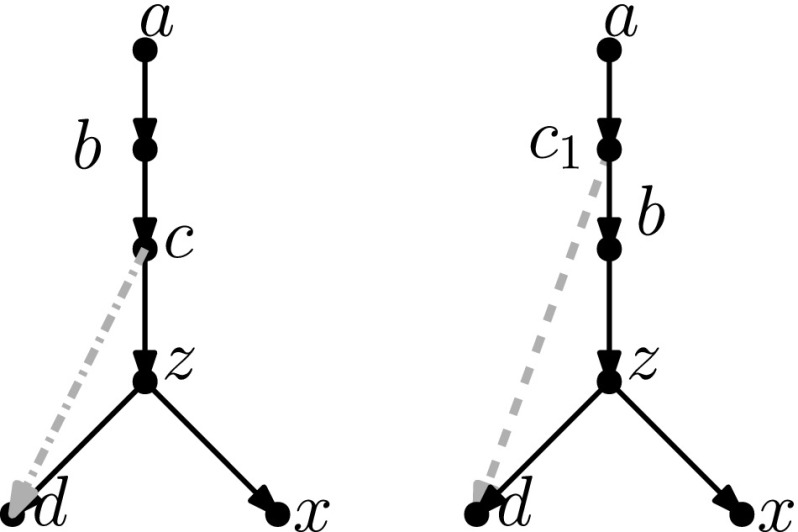
(Color figure online) Proof of Lemma 4.6, Case 1(b)iiA: If (*z*, *x*) is not movable because of the triangle with long edge (*c*, *d*), and the parent of *c* is not the root of *N*, then we move the tail of (*c*, *d*) ‘further up’ in order to make (*z*, *x*) movable

**Fig. 15:**
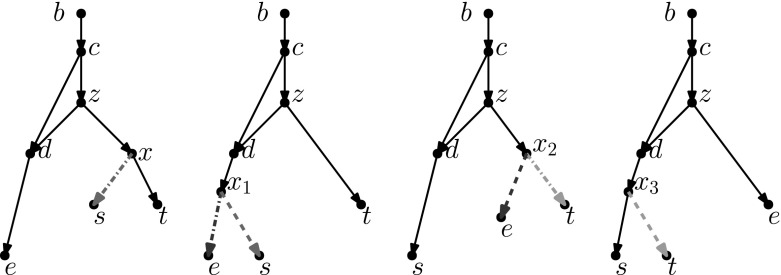
(Color figure online) Proof of Lemma 4.6, Case 1(b)iiB: Note that the set of tail moves depicted is equivalent to moving the head of (*c*, *d*) to (*z*, *x*). After this sequence of moves, *x* now has a reticulation parent

1. **There exists a lowest node** $\varvec{u}'$ **of** $\varvec{N' \setminus Y'}$ **such that** $\varvec{u'}$ **is a reticulation** In this case, let $x'$ be the single child of $u'$. Then, $x'$ is in $Y'$, and therefore there exists a node $x \in Y$ such that *x* is mapped to $x'$ by the isomorphism between *N*[*Y*] and ${N'[Y']}$. Furthermore, *x* has the same number of parents in *N* as $x'$ does in $N'$ (because the networks are binary and *x* has the same number of children in *N* as $x'$ does in $N'$ by the isomorphism between *N*[*Y*] and ${N'[Y']}$), and the same number of parents in *Y* as $x'$ has in $Y'$. Thus, *x* has at least one parent *z* such that *z* is not in *Y*.
  We now split into two subcases:
  - $\varvec{z}$ **is a reticulation** in this case, let $Y_1 = Y \cup \{z\}$ and $Y_1' = Y' \cup \{u'\}$, and extend the isomorphism between *N*[*Y*] and ${N'[Y']}$ to an isomorphism between $N[Y_1]$ and ${N'[Y_1']}$, by letting *z* be mapped to $u'$ (see Fig. 12). We now have that $Y_1$ and $Y_1'$ are downward-closed sets of nodes such that $N[Y_1]$ is isomorphic to $N[Y_1']$, and $L(N)\subseteq Y_1, L(N') \subseteq Y_1'$. Furthermore, $|N \setminus Y_1| = |N \setminus Y|-1$. Thus, by the inductive hypothesis, there is a sequence of $3|N \setminus Y_1| = 3|N \setminus Y|-3$ tail moves turning *N* into $N'$.
  - $\varvec{z}$ **is not a reticulation** then *z* cannot be the root of *N* (as this would imply $|N \setminus Y| = 1$), so *z* is a tree node. It follows that the edge (*z*, *x*) is movable, unless the removal of (*z*, *x*) followed by suppressing *z* creates parallel edges.
    (i) $\varvec{(z,x)}$ **is movable** In this case, let *u* be any reticulation in $N \setminus Y$ (such a node must exist, as $u'$ exists and $N \setminus Y$, $N' \setminus Y'$ have the same number of reticulations). Let *v* be the child of *u* (which may be in *Y*), and observe that the edge (*u*, *v*) is not below *x* (as $x \in Y$ and $u \notin Y$). If $v = x$, then *u* is a reticulation parent of *x* that is not in *Y*, and by substituting *v* for *z*, we have case 1a. So we may assume $v \ne x$. Then, it follows from Observation 2.11 that the tail of (*z*, *x*) can be moved to (*u*, *v*). Let $N_1$ be the network derived from *N* by applying this tail move, and let $z_1$ be the new node created by subdividing (*u*, *v*) during the tail move (see Fig. 13).
      Thus, $N_1$ contains the edges $(u,z_1), (z_1,v), (z_1,x)$. (Note that if *z* is immediately below *u* in *N* i.e. $z=v$, then in fact $N_1 = N$. In this case, we may skip the move from *N* to $N_1$, and in what follows substitute *z* for $z_1$.)
      Note that $(z_1,v)$ is movable in $N_1$, since the parent *u* of *z* is a reticulation node, and therefore deleting $(z_1,v)$ and suppressing $z_1$ cannot create parallel edges. Let *w* be one of the parents of *u* in $N_1$. Then, the tail of $(z_1,v)$ can be moved to (*w*, *u*) (as $u \ne v$, and (*w*, *u*) is not below *v* as this would imply a cycle in $N_1$).
      So now let $N_2$ be the network derived from $N_1$ by applying this tail move (again see Fig. 13). In $N_2$, the reticulation *u* is the parent of *x* (as $z_1$ was suppressed), and thus Case 1a applies to $N_2$ and $N'$. Therefore, there exists a sequence of $3|N \setminus Y|-3$ tail moves turning $N_2$ into $N'$. As $N_2$ is derived from *N* by two tail moves, there exists a sequence of $3|N \setminus Y|-1$ tail moves turning *N* into $N'$.
    (ii) **The removal of** $\varvec{(z,x)}$ **followed by suppressing** $\varvec{z}$ **creates parallel edges** Then, there exists nodes $c \ne x, d \ne x$ such that *c*, *d*, *z* form a triangle with long edge (*c*, *d*). As *c* has outdegree 2 it is not the root of *N*, so let *b* denote the parent of *c*.
      (A) $\varvec{b}$ **is not the root of** $\varvec{N}$ In this case, let *a* be a parent of *b* in *N*. Observe that the edge (*a*, *b*) is not below *d* and that $b \ne d$. Furthermore, (*c*, *d*) is movable since *c* is not a reticulation or the root. Moreover, there is no edge (*b*, *z*): The existence of such an edge would imply that *z* has indegree 2, as the edge (*c*, *z*) exists, but this is not possible because *z* is a tree node with child edges (*z*, *x*) and (*z*, *c*). It follows that the tail of (*c*, *d*) can be moved to (*a*, *b*) (again using Observation 2.11). Let $N_1$ be the network derived from *N* by applying this tail move (see Fig. 14).
        Observe that in $N_1$ we now have the edge (*b*, *z*) (as *c* was suppressed), and still have the edge (*z*, *d*) but not the edge (*b*, *d*) (such an edge would mean *d* has indegree 3, as the edges (*c*, *d*) and (*z*, *d*) exist). Thus, deleting (*z*, *x*) and suppressing *z* will not create parallel edges, and so (*z*, *x*) is movable in $N_1$. Thus Case 1(b)i applies to $N_1$ and $N'$, and so there exists a sequence of $3|N \setminus Y|-1$ tail moves turning $N_1$ into $N'$. As $N_1$ is derived from *N* by a single tail move, there exists a sequence of $3|N \setminus Y|$ tail moves turning *N* into $N'$.
      (B) $\varvec{b}$ **is the root of** $\varvec{N}$ In this case, we observe that if $d \in Y$ then every reticulation in *N* is in *Y*. This contradicts the fact that $N\setminus Y$ and $N'\setminus Y'$ contain the same number of reticulations. Therefore, we may assume that $d \notin Y$. Then, we may proceed as follows. Let $N_3$ be the network derived from *Y* by moving the *head* of (*c*, *d*) to (*z*, *x*). As this is a head move of type (a), it can be replaced with a sequence of four tail moves (see Fig. 7). However, we show that in this particular case, it is possible to replace it with a sequence of only three tail moves.
        Let *e* be the child of *d* in *N*. We note that if *e* is a reticulation, then one of the parents of *e* is a descendant of *x* (otherwise *z*, *b* or *c* would have to be a parent of *e*, which is not the case). Thus if *e* is a reticulation then it is a descendant of *x*, and by a similar argument if *x* is a reticulation then it is a descendant of *e*. Thus, we may assume that one of *e*, *x* is not a reticulation, and that furthermore at least one of *e*, *x* is a tree node (if both are leaves, then *N* and $N'$ are the network depicted in the second part of Fig. 8 and the network resulting from applying head move (a) in this network; and if one of *e* and *x* is a reticulation, then the other must be an ancestor of it, and is therefore a tree node).
        Our approach in this case will be to “swap” the positions of *e* and *x* via a series of tail moves. We will assume that *x* is a tree node, with children *s* and *t*. (The case that *e* is a tree node can be handled in a similar manner.) Then we apply the sequence of tail moves depicted in Fig. 15.
        Observe that in $N_3$, *x* has a parent not in *Y* which is a reticulation, and that $N_3[Y] = N[Y]$. Then Case 1a applies to $N_3$ and $N'$, and so there exists a sequence of $3|N \setminus Y|-3$ tail moves turning $N_3$ into $N'$. As $N_3$ can be derived from *N* by a sequence of 3 tail moves, it follows that there exists a sequence of $3|N \setminus Y|$ tail moves turning *N* into $N'$.
  **Fig. 13:**
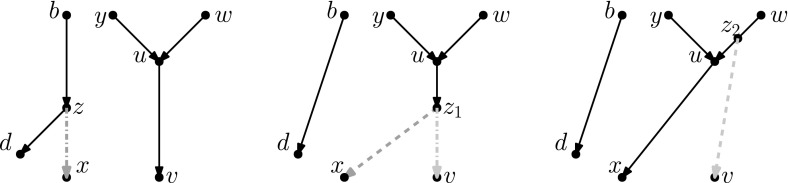
(Color figure online) Proof of Lemma 4.6, Case 1(b)i: If (*z*, *x*) is movable, we may move the tail of (*z*, *x*) to (*u*, *v*) so that the parent $z_1$ of *x* is below *u*, then move the tail of $(z_1,v)$ so that the reticulation *u* becomes a parent of *x*

**Fig. 16:**
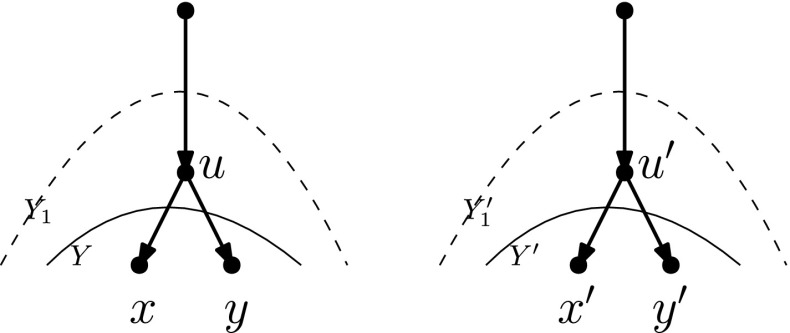
(Color figure online) Proof of Lemma 4.6, Case 3a: If $u'$ is a lowest reticulation in $N' \setminus Y'$ with children $x',y'$, and the nodes $x,y \in Y$ corresponding to $x',y'$ share a reticulation parent *u* in $N \setminus Y$, then we may add *u* to *Y* and $u'$ to $Y'$
2. **There exists a lowest node** $\varvec{u}$ **of** $\varvec{N \setminus Y}$ **such that** $\varvec{u}$ **is a reticulation** By symmetric arguments to Case 1, we have that there is a sequence of at most $3|N\setminus Y|$ tail moves turning $N'$ into *N*. As all tail moves are reversible, there is also a sequence of at most $3|N\setminus Y|$ tail moves turning *N* into $N'$.
3. **No lowest node of** $\varvec{N \setminus Y}$ **nor any lowest node of** $\varvec{N'\setminus Y'}$ **is a reticulation** As $|N \setminus Y| > 1$, we have that in fact every lowest node of $N \setminus Y$ and every lowest node of $N'\setminus Y'$ is a tree node. Then, we proceed as follows. Let $u'$ be an arbitrary lowest node of $N' \setminus Y'$, with $x'$ and $y'$ its children.
  Then $x',y'$ are in $Y'$, and therefore there exist nodes $x,y \in Y$ such that *x* (*y*) is mapped to $x'$ ($y'$) by the isomorphism between *N*[*Y*] and ${N'[Y']}$. Furthermore, *x* has the same number of parents in *N* as $x'$ does in $N'$, and the same number of parents in *Y* as $x'$ has in $Y'$ (again because the networks are binary and *x* has the same number of children in *N* as $x'$ does in $N'$ by the isomorphism between *N*[*Y*] and ${N'[Y']}$). Thus, *x* has at least one parent not in *Y*. Similarly, *y* has at least one parent not in *Y*.
  - $\varvec{x}$ **and** $\varvec{y}$ **have a common parent** $\varvec{u}$ **not in** $\varvec{Y}$ In this case, let $Y_1 = Y \cup \{u\}$ and $Y_1' = Y' \cup \{u'\}$, and extend the isomorphism between *N*[*Y*] and ${N'[Y']}$ to an isomorphism between $N[Y_1]$ and ${N'[Y_1']}$, by letting *u* be mapped to $u'$ (see Fig. 16). We now have that $Y_1$ and $Y_1'$ are downward-closed sets of nodes such that $N[Y_1]$ is isomorphic to ${N'[Y_1']}$, and $L(N)\subseteq Y_1, L(N') \subseteq Y_1'$. Furthermore, $|N \setminus Y_1| < |N \setminus Y|$. Thus by the inductive hypothesis, there is a sequence of $3|N \setminus Y_1| = 3|N \setminus Y|-3$ tail moves turning *N* into $N'$.
  - $\varvec{x}$ **and** $\varvec{y}$ **do not have a common parent not in** $\varvec{Y}$ In this case, let $z_x$ be a parent of *x* not in *Y*, and let $z_y$ be a parent of *y* not in *Y*. Recall that $z_x$ and $z_y$ are both tree nodes. It follows that either one of $(z_x,x), (z_y,y)$ is movable, or deleting $(z_x,x)$ and suppressing *x* (deleting $(z_y,y)$ and suppressing *y*) would create parallel edges.
    (i) $\varvec{(z_x,x)}$ **is movable** in this case, observe that the edge $(z_y,y)$ is not below *x* (as $x \in Y$ and $z_y \notin Y$), and that $x \ne y$. Then, by Observation 2.11, the tail of $(z_x,x)$ can be moved to $(z_y,y)$.
      Let $N_1$ be the network derived from *N* by applying this tail move (see Fig. 17). Then, as *x* and *y* have a common parent in $N_1$ not in *Y*, and as $N_1[Y] = N[Y]$, we may apply the arguments of Case 3a to show that there exists a sequence of $3|N \setminus Y|-3$ tail moves turning $N_1$ into $N'$. As $N_1$ is derived from *N* by a single tail move, there exists a sequence of $3|N \setminus Y|-2$ tail moves turning *N* into $N'$.
    (ii) $\varvec{(z_y,y)}$ **is movable** By symmetric arguments to Case 3(b)i, we have that there is a sequence of at most $3|N \setminus Y|-2$ tail moves turning *N* into $N'$.
    (iii) **Neither** $\varvec{(z_x,x)}$ **nor** $\varvec{(z_y,y)}$ **is movable** In this case, there must exist nodes $d_x,c_x, d_y, c_y$ such that $c_x,d_x,z_x$ form a triangle with long edge $(c_x,d_x)$, and $c_y,d_y,z_y$ form a triangle with long edge $(c_y,d_y)$. Moreover, as $z_x,z_y$ are different nodes with one parent each, $c_x \ne c_y$. It follows that one of $c_x,c_y$ is not the child of the root of *N*. Suppose without loss of generality that $c_x$ is not the child of the root. Then, there exist nodes $a_x,b_x$ and edges $(a_x,b_x), (b_x,c_x)$.
      By similar arguments to those used in Case 1(b)iiA, the tail of $(c_x,d_x)$ can be moved to $(a_x,b_x)$, and in the resulting network $N_1$, $(z_x,x)$ is movable (see Fig. 18). Thus, Case 3(b)i applies to $N_1$ and $N'$, and so there exists a sequence of $3|N\setminus Y|-2$ tail moves turning $N_1$ into $N'$. As $N_1$ is derived from *N* by a single tail move, there exists a sequence of $3|N\setminus Y|-1$ tail moves turning *N* into $N'$.

$$\square $$

**Fig. 17:**
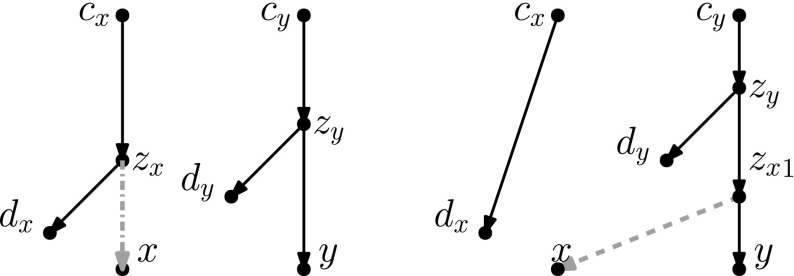
(Color figure online) Proof of Lemma 4.6, Case 3(b)i: If $(z_x,x)$ is movable, we may move the tail of $(z_x,x)$ to $(z_y,y)$ so that *x* and *y* share a parent

**Fig. 18:**
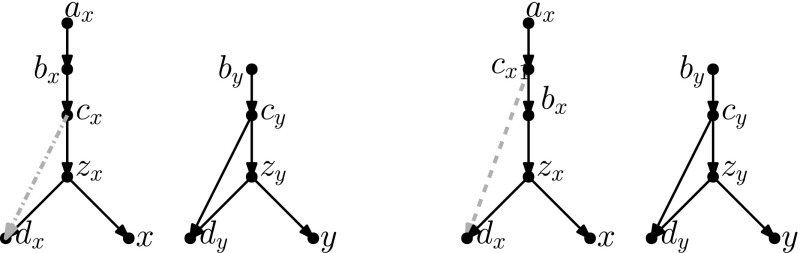
(Color figure online) Proof of Lemma 4.6, Case 3(b)iii: If neither $(z_x,x)$ or $(z_y,y)$ is movable, we can make at least one of them movable by moving the long edge of its triangle “further up”

By setting $Y = L(N)$ and $Y' = L(N')$, we have the following:

#### Theorem 4.7

Let *N* and $N'$ be networks in the *k*th tier on *X* such that *N* and $N'$ are not the networks depicted in Fig. 8. Then, there is a sequence of at most $3(|N|-|X|)=3(|X|+2k)$ tail moves turning *N* into $N'$.

As rSPR moves consist of head moves and tail moves, Theorem 4.7 also gives us an upper bound on the number of rSPR moves needed to turn *N* into $N'$. By modifying these arguments slightly, we can improve this bound in the case of rSPR moves.

#### Theorem 4.8

Let *N* and $N'$ be networks in the *k*th tier on *X*. Then, there is a sequence of at most $2|X|+3k-1$ rSPR moves turning *N* into $N'$.

#### Proof

Recall that the proof of Lemma 4.6 works by gradually expanding two downward-closed subsets $Y \subseteq V(N), Y' \subseteq V(N')$ for which *N*[*Y*] is isomorphic to ${N'[Y']}$, using at most 3 tail moves each time the size of *Y* and $Y'$ is increased. We show that in Cases 1 and 2 of the proof of Lemma 4.6, we may instead use one head move. Indeed, in Case 1 there is a lowest node $u'$ of $N'\setminus Y'$ that is a reticulation with child $x' \in Y$, and the node $x \in Y$ corresponding to $x'$ has parent *z*. If *z* is a reticulation (Case 1a), then as before there is no need for any move, we simply add *z* to *Y* and $u'$ to $Y'$. If *z* is not a reticulation, we proceed as follows. There exists some reticulation node in $v \in N\setminus Y$ (again, such a node must exist, as $u'$ exists and $N\setminus Y$ and $N'\setminus Y'$ have the same number of reticulations). Moving one of its parent edges (*u*, *v*) to (*z*, *x*) will not create a cycle, as *Y* is downward-closed. It cannot create any parallel edges, unless either $u = z$, or the other parent edge (*w*, *v*) is part of a triangle on *w*, *v* and the child of *v*. If $u = z$, then we can move (*w*, *v*) to (*z*, *x*) and this will not create parallel edges unless *z*, *v*, *x* form a triangle. But in this case, *x* is a child of *v* and thus *x* already has a parent in $N \setminus Y$ that is a reticulation. This implies that, after at most one head move, *x* has a reticulation parent, and we may proceed as in Case 1a. (Case 2 is handled symmetrically.)

The other cases use at most one tail move (and thus at most one rSPR move), apart from Case 3(b)iii that may require 2 rSPR moves. This case can come up as many times as there are tree nodes in the network. Hence, the number of moves needed to add a node to *Y* is at most one for each reticulation node, and at most two for each tree node. This means at most $|V|-|X|+t$ moves are needed, where *t* denotes the number of tree nodes in *N* (and thus in $N'$).

Recall from Observation 2.4 that $|V|=2(|X|+k)$ for any binary tier *k* network. As *k* nodes are reticulations, |*X*| are leaves and 1 is the root, we have $t = |X|+k-1$. This shows that we need at most $2(|X|+k)-|X|+|X|+k-1=2|X|+3k-1$ rSPR moves to turn *N* into $N'$. $\square$

In practice, we expect the distance between most pairs of networks to be less than $|X|+3k-1$ because only one case needs two rSPR moves, and this case might not come up very often.

The next observation will be useful for obtaining lower bounds for the diameter of tail and rSPR moves.

#### Observation 4.9

Let *N* and $N'$ be networks in the *k*th tier on *X*, and let $n:=|X|$ be the number of leaves. The observation that $\mathrm{Tail}_1\subseteq \mathrm{rNNI}\subseteq \mathrm{rSPR}$ (where $\mathrm{Tail}_1$ denotes the class of distance-1 tail moves) implies that

$$\begin{aligned} d_{\mathrm{Tail}_1}(N,N')\ge d_{\mathrm{rNNI}}(N,N')\ge d_{\mathrm{rSPR}}(N,N'), \end{aligned}$$

and

$$\begin{aligned} \varDelta _k^{\mathrm{Tail}_1}(n)\ge \varDelta _k^{\mathrm{rNNI}}(n)\ge \varDelta _k^{\mathrm{rSPR}}(n). \end{aligned}$$

Similarly, $\mathrm{Tail}_1\subseteq \mathrm{Tail}\subseteq \mathrm{rSPR}$ implies that

$$\begin{aligned} d_{\mathrm{Tail}_1}(N,N')\ge d_{\mathrm{Tail}}(N,N')\ge d_{\mathrm{rSPR}}(N,N'), \end{aligned}$$

and

$$\begin{aligned} \varDelta _k^{\mathrm{Tail}_1}(n)\ge \varDelta _k^{\mathrm{Tail}}(n)\ge \varDelta _k^{\mathrm{rSPR}}(n). \end{aligned}$$

Diameters of move-induced metrics on tree space are well studied. A few relevant bounds are $\varDelta _0^{\mathrm{rSPR}}=n-\varTheta (\sqrt{n})$ (Ding et al. 2011; Atkins and McDiarmid 2015) and $\varDelta _0^{\mathrm{rNNI}}=\varTheta (n\log (n))$ (Li et al. 1996). We extend these results to higher tiers of network space.

Lemma 4.5 gives lower bounds of order $n-\varOmega (\sqrt{n})$ on diameters for rSPR and tail moves, by reducing to trees and using the corresponding diameter bound. Theorem 4.7 and Theorem 4.8 give upper bounds of order *n*. More precisely, $\varDelta _k^{\mathrm{Tail}}(n)\le 3(n+2k)$ and $\varDelta _k^{\mathrm{rSPR}}(n)\le 2n+3k$ from Observation 4.9. The following theorem summarizes this discussion:

#### Theorem 4.10

The diameter of tiers of network space for metrics induced by rSPR and tail moves satisfy

$$\begin{aligned} \begin{aligned} n-\varTheta (\sqrt{n})&\le \varDelta _k^{\mathrm{Tail}}(n)\le 3(n+2k),\\ n-\varTheta (\sqrt{n})&\le \varDelta _k^{\mathrm{rSPR}}(n)\le 2n+3k. \end{aligned} \end{aligned}$$

Additionally, going back through the proof of Theorem 3.4, we see that any distance one head move can be replaced by at most four tail moves if the network has more than one leaf, so $d_{\mathrm{Tail}}(N,N')\le 4 d_{\mathrm{rNNI}}(N,N')$ for any pair of networks $N,N'$ with more than one leaf. Note that this does not give us new information regarding the diameters, but it does give bounds on the distances when we are given two networks.

### The Diameter of Tail_1_ and rNNI Moves

Comparing rNNI and tail moves directly is complicated by the fact that rNNI moves are more local. To make the comparison easier, we consider local tail moves: tail moves over small distance. The following lemma indicates how restricting to distance-1 tail moves influences our results.

#### Lemma 4.11

Let $e=(u,v)$ to $f=(s,t)$ be a valid tail move in a tier *k* network *N* on *X*. Then, there is a sequence of at most $|X|+3k-1$ distance-1 tail moves resulting in the same network.

#### Proof

Note that there exist directed paths ${{\mathrm{LCA}}}(u,s)\rightarrow ~s$ and ${{\mathrm{LCA}}}(u,s)\rightarrow ~u$ (which are not necessarily unique) for any choice of ${{\mathrm{LCA}}}(u,s)$. We prove that a tail move of *e* to any edge on either path is valid, and this gives a sequence of distance-1 tail moves: Indeed, for any edges *f* and *g* that share a node, if there is a valid tail move *e* to *f* resulting in network $N_f$, and a valid tail move *e* to *g* resulting in network $N_g$, then there is a distance-1 tail move *f* to *g* that transforms $N_f$ into $N_g$, and furthermore as $N_g$ has no cycles or parallel edges, this is a valid tail move.

Let $g=(x,y)$ be an edge of one of these two paths. We first use a proof by contradiction to show that the move to *g* cannot create cycles, then we prove that we do not create parallel edges.

Suppose moving *e* to *g* creates a cycle, then this cycle must involve the new edge $e'$ from *g* to *v*. This means there is a path from *v* to *x*. However, *x* is above *u* or above *s*, which means that either the starting graph is not a phylogenetic network, or the move *e* to *f* is not valid. From this contradiction, we conclude that the move of *e* to *g* does not create cycles.

Note that *e* is movable, because the move of *e* to *f* is valid. Hence, the only way to create parallel edges is by moving $e=(u,v)$ to an edge $g=(x,y)$ with $y=v$. It is clear that *g* is not in the path ${{\mathrm{LCA}}}(u,s)\rightarrow u$, as this would imply the existence of a cycle in the original network. Hence, *g* must be on the other path. If $f=g$, then the original move of *e* to *f* would create parallel edges, and if *g* is above *f*, the original move moves *e* to below *e* creating a cycle. We conclude that there cannot be an edge $g=(x,y)$ on either path such that $y=v$; hence, we do not create parallel edges.

Noting that a path between two nodes uses at most $|E|-|X|$ edges, we see that we need at most $|E|-|X|=|X|+3k-1$ distance-1 tail moves to simulate a long distance tail move (the last equivalence holds by Observation 2.4). $\square$

Note that a distance-*d* tail move cannot necessarily be simulated with a sequence of *d* distance-1 tail moves. The path of length *d* defining the distance of the tail move might not be a path over which we can move the tail, if for example some of its edges are below the head.

Lemma 4.11 directly gives us upper bounds on $\hbox {Tail}_1$ and rNNI diameters in terms of the tail diameter: each tail move is replaced by distance-1 tail moves, giving an upper bound of $(3n+6k)(n+3k-1)$ for tier *k* networks on *n* leaves. As we are mostly interested in the effect of the number of leaves, we denote these bounds $\varDelta _k^{\text {Tail}_1}(n)=O(n^2)$ and $\varDelta _k^{\mathrm{rNNI}}(n)=O(n^2)$ (because $\text {Tail}_1\subseteq \text {rNNI}$).

Francis et al. (2017) proved diameter bounds for $d_{\mathrm{NNI}}$ on *unrooted* networks. These moves do not have to account for the orientation of edges. Therefore, they generally define larger classes of moves. More precisely, given a rooted network *N* with unrooted underlying graph *U*(*N*), the set of unrooted networks that can be reached with one NNI move from *U*(*N*) contains the set of unrooted networks we get by applying one rNNI move to *N* and then taking the underlying graph. Francis et al. give the following lower bound on NNI diameters using Echidna graphs (Francis et al. 2017, Theorem 4.3):

$$\begin{aligned} \left[ (v_k^n-3)\log _6\left( \frac{v_k^n}{2}-2\right) -(2k-1)\log _6(k-1)-\left( v_k^n-2k\right) \log _6 e -2v_k^n\right] , \end{aligned}$$

where $v_k^n$ is the number of nodes in an unrooted network with $n+1$ leaves and *k* reticulation nodes. This lower bound is $\varOmega (n \log (n))$ for fixed *k*. As Echidna graphs are rootable (i.e., for each Echidna graph there exists some rooted network with this Echidna graph as underlying unrooted network), the argument of Francis et al. easily extends to rooted networks and we get the same lower bound for $\varDelta _k^{\mathrm{rNNI}}(n)$. The preceding discussion proves the following theorem:

#### Theorem 4.12

The diameter of tiers of network space for metrics induced by rNNI and distance-1 tail moves satisfy

$$\begin{aligned}&\varDelta _k^{\mathrm{rNNI}}(n)=\varOmega (n \log (n))\quad \mathrm{and }\quad \varDelta _k^{\mathrm{rNNI}}(n)=O(n^2),\\&\varDelta _k^{\mathrm{Tail}_1}(n)=\varOmega (n \log (n))\quad \mathrm{and }\quad \varDelta _k^{\mathrm{Tail}_1}(n)=O(n^2). \end{aligned}$$

### The Diameter of SPR Moves

In this subsection, we will give a new upper bound on the diameter of SPR moves on unrooted networks, using results for rSPR moves on rooted networks. This linear bound improves on the previously best quadratic bound: $(v_k^n)^2+4v_k^n$, where $v_k^n$ denotes the number of nodes in a tier *k* network with *n* leaves (Francis et al. 2017).

The underlying unrooted network of a rooted network *N* is denoted *U*(*N*). An unrooted network *U* is called *rootable* if there exists a rooted network *N* such that $U(N)=U$. Note that as the root *r* of *N* has out degree 1 in *N*, *r* will be a leaf in *U*. As mentioned at the end of Sect. 2.2, not all unrooted networks are rootable (see Fig. 4).

Any edge of an unrooted network whose removal disconnects the network is called a *cut-edge*. If only one of the components contains leaves, the edge is called a *redundant cut-edge*. A *blob* of an unrooted network is a non-trivial biconnected component, i.e. a maximal subgraph with at least two vertices and no cut-edges. The next lemma characterizes rootable networks via redundant cut-edges:

#### Lemma 4.13

An unrooted network is rootable if and only if it has no redundant cut-edges.

#### Proof

First let *U* be some unrooted network with no redundant cut-edges. To show that *U* is rootable, we pick any leaf *r* of *U* and show how to construct a rooted network with *U* as underlying graph and *r* as root. First, orient all cut-edges “away” from *r*. Then, it only remains to find a valid orientation of every blob. To this end, let *B* be a blob. After orienting all cut-edges, *B* has only one incoming edge (*s*, *t*) and, as (*s*, *t*) is not redundant, *B* has at least one outgoing edge (*x*, *y*). Since *B* is biconnected, there is a bipolar (i.e. acyclic) orientation of *B* with *t* as source and *x* as sink (Lempel et al. 1967). Doing the same for all biconnected components, we get an acyclic orientation of *U* rooted at *r*.

Conversely, suppose that *U* has a redundant cut-edge *e*. Deleting *e* creates a component *C* without leaves. If we direct *e* towards *C*, then *C* has one source and no possible sinks (no leaves). If we direct *e* away from *C*, then *C* has one sink but no possible sources (since the root is also a leaf). This implies there is no valid orientation of the edges in *C* and therefore in *U*. $\square$

A *redundant terminal component* of an unrooted network *U* is a non-trivial biconnected component that is incident to exactly one cut-edge (which must be a redundant cut-edge). The next lemma, which follows directly from Lemma 4.13, characterizes rootable networks via redundant terminal components.

#### Lemma 4.14

An unrooted network is rootable if and only if it has no redundant terminal components.

We now give a formal definition of a SPR move:

#### Definition 4.15

Let *U* be an unrooted network, and let $\{u,v\}$, $\{x,u\}$, $\{u,y\}$ and *e* be edges of *U*. The *SPR move* of the *u*-end of $\{u,v\}$ to *e* consists of the subdivision of *e* with $u'$, the removal of $\{u,v\}$, the suppression of *u*, and the addition of edge $\{u',v\}$. The move is only valid if the resulting graph is an unrooted network, i.e. if it is connected and has no parallel edges.

The next two lemmas give upper bounds for the number of redundant terminal components in an unrooted network with reticulation number $k=|E|-|V|+1$, and for the number of SPR moves needed to get to an unrooted network without redundant terminal components.

#### Lemma 4.16

Let *U* be an unrooted network in the *k*th tier. Then *U* has at most *k* / 3 redundant terminal components.

#### Proof

Let $V'$ be the nodes in a redundant terminal component, and $E'$ the edges with at least one endpoint in $V'$. Then, every node in $V'$ has degree 3 with respect to $E'$ and every edge in $E'$ except one has both endpoints in $V'$, which implies that $3|V'| = 2|E'|- 1$. It follows that $|V'|$ is odd. Furthermore, $V'$ must be greater than 3 in order for every node to have degree 3. Thus, $|V'|\ge 5$, and hence $|E'|-|V'| = (3|V'|+1)/2 - |V'| = (|V'|+1)/2 \ge (5+1)/2 = 3$. Now observe that every node and edge of the network appear in at most one such set $V'$ or $E'$. Furthermore, if all nodes and edges in all such sets $V'$ and $E'$ are deleted, the resulting graph $(V'',E'')$ is still connected because only redundant terminal components are removed. Hence, the remaining part satisfies $|E''| - |V''| \ge -1$. Note that $|E| - |V|$ is equal to $|E''|-|V''|$ plus the sum of all $|E'|-|V'|$ for every redundant terminal component with nodes $V'$ and incident edges $E'$. Thus, if *U* has *t* redundant terminal components, then

$$\begin{aligned} |E|-|V| ={ |E''|-|V''| + \sum _{i=1}^{t}\Big (|E'_i|-|V'_i|\Big ) \ge |E''|-|V''| + 3t \ge 3t-1,} \end{aligned}$$

where $V_i$ and $E_i$ denote the nodes and the edges of the *i*th redundant terminal component of *U*. It follows that if *U* has more than $t=k/3$ redundant terminal components, then the reticulation number of *U* is $|E|-|V|+1 > 3(k/3)$, a contradiction. $\square$

#### Lemma 4.17

Let *N* be an unrooted network in the *k*th tier with *c* redundant terminal components. Then, there exists an unrooted network $N'$ in the *k*th tier with at most $c-1$ redundant terminal components such that $d_{\mathrm{SPR}}(N,N')=1$.

#### Proof

**Fig. 19:**
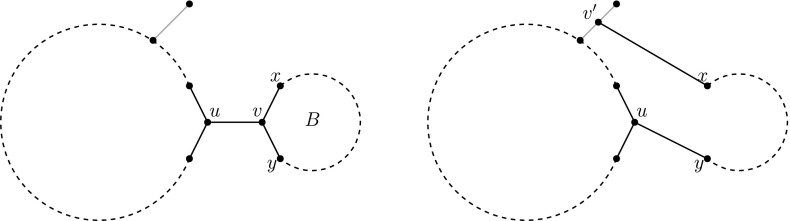
The SPR move used to remove redundant terminal component *B*. The big circle represents all of *U* except for *B*. Note that in the unrooted network on the right, *B* is not a redundant terminal component anymore, and no extra redundant terminal components have been created

Pick any redundant terminal component *B* and let $\{u,v\}$ be the unique edge for which $u \notin B$, $v \in B$. Let *x* and *y* be the other neighbours of *v*. Now SPR move the *v*-end of edge $\{v,x\}$ to a leaf edge of *U*. Suppressing *v* cannot give parallel edges, because $\{u,v\}$ is a cut-edge (Fig. 19). In the resulting unrooted network, *B* is extended to a biconnected component with a pendant leaf, and because no new cut-edges have been created, the network has at most $c-1$ redundant terminal components and is one SPR move away from the original unrooted network. Note that the networks are in the same tier because an SPR move does not change the number of edges nor the number of vertices. $\square$

Lemmas 4.14, 4.16 and 4.17 imply the following:

#### Corollary 4.18

Any tier *k* unrooted network is at most *k* / 3 SPR moves away from a rootable network.

The next two results relate a set of rSPR moves between two rooted networks and the corresponding set of SPR moves between their underlying unrooted networks.

#### Lemma 4.19

Any rSPR move transforming a rooted network *N* into another rooted network $N'$ has a corresponding SPR move transforming *U*(*N*) into $U(N')$.

#### Proof

Suppose that the rSPR move is a head move of (*u*, *v*) to *e*. Then, $U(N')$ is the unrooted network we get by doing the SPR move of the *v*-end of $\{u,v\}$ to *e*. This SPR move is valid because $N'$, and therefore $U(N')$ is connected with no parallel edges. Similarly, a tail move of (*u*, *v*) to *e* has a corresponding SPR move moving the *u*-end of $\{u,v\}$ to *e*. $\square$

#### Corollary 4.20

Any rSPR sequence transforming a rooted network *N* into another rooted network $N'$ has a corresponding SPR sequence transforming *U*(*N*) into $U(N')$.

Moreover, since any rootable network is the underlying graph *U*(*N*) of some rooted network *N*, the diameter for rSPR on rooted networks gives an upper bound for the diameter of SPR moves on unrooted rootable networks.

We do not apply the following proposition here, but it may become useful when a better bound on $\varDelta _k^{\text {rSPR}}(n)$ is found in future research.

#### Proposition 4.21

The diameter of the *k*th tier of unrooted network space has upper bound

$$\begin{aligned} \varDelta _k^{\mathrm{SPR}}(n)\le \varDelta _k^{\mathrm{rSPR}}(n)+2M, \end{aligned}$$

where *M* denotes the maximal SPR distance from any unrooted network to a rootable network.

#### Proof

Let *N* and $N'$ be tier *k* unrooted networks. Then, we can do *M* moves from *N* and *M* moves from $N'$ to get to rootable networks $N_r$ and $N'_r$. Choose a root for both these rootable networks, then by Corollary 4.20 there is a sequence of at most $\varDelta _k^{\text {rSPR}}(n)$ moves to go from $N_r$ to $N'_r$. Because all moves are reversible, there is a sequence of moves:of length at most $\varDelta _k^{\text {rSPR}}(n)+2M$ from *N* to $N'$. $\square$

This proposition together with Corollary 4.18 and Theorem 4.8 gives a reasonable bound on the diameter of unrooted networks, namely $2|X|+3k+\frac{2}{3}k$. However, using the (lack of) structure in an unrooted network, we can do better. The next theorem again uses rooted networks, but dynamically re-orientates the network during the induction.

#### Theorem 4.22

The SPR diameter of the *k*-tier of unrooted network space has upper bound

$$\begin{aligned} \varDelta _k^{\mathrm{SPR}}(n) \le n+\frac{8}{3}k. \end{aligned}$$

#### Proof

Let *N* and $N'$ be tier *k* unrooted networks. As before, we use $\frac{2}{3}k$ moves to produce rootable networks $N_r$ and $N'_r$. Now we do induction as in the proof of the rooted rSPR diameter (Theorem 4.8). Choose a network orientation on both rootable networks. As before, we construct the downward-closed subsets *Y* and $Y'$ of $N_r$ and $N'_r$ such that $N_r|_{Y}$ and $N'_r|_{Y'}$ are isomorphic. We prove that we need at most $n+2k$ moves in total to produce the rootable network $N'_r$ from $N_r$ by inductively increasing the size of *Y* and $Y'$.

As in the proof of Theorem 4.8, we can add a node to *Y* and $Y'$ using at most one move, except in the case that all lowest nodes are tree nodes and the nodes corresponding to their child nodes have unmovable incoming edges. We now show how this case can be treated differently if we consider SPR moves on unrooted networks.

**Fig. 20:**
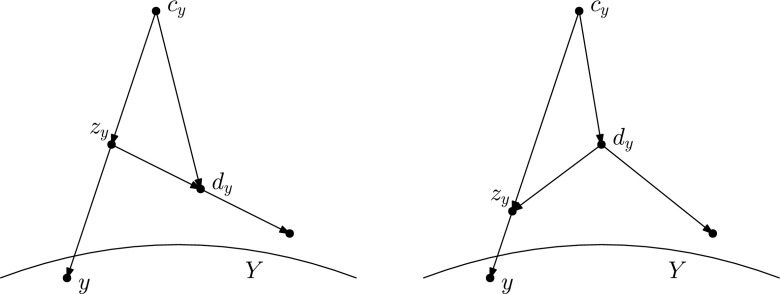
The re-orientation of the bottom edge of a triangle, all of whose nodes are above *Y*

**Fig. 21:**
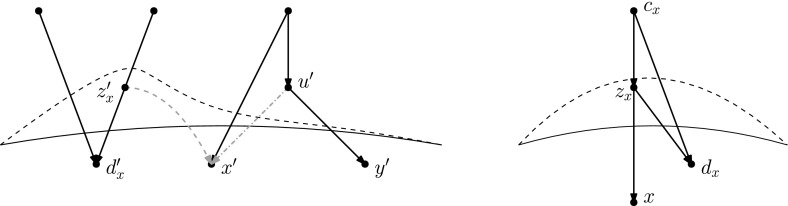
(Color figure online) The tail move used in the proof of Case 3a in the proof of Theorem 4.22

Suppose we are in the situation of Case 3(b)iii. We shortly recall the situation: *Every lowest node of* $N \setminus Y$ *and every lowest node of* $N'\setminus Y'$ *is not a reticulation, so there exists a node* $u'\in N'\setminus Y'$ *with children* $x',y'\in Y'$. *Let* *x*, *y* *be the nodes in* *Y* *corresponding to* $x'$ *and* $y'$. *The nodes* *x* *and* *y* *do not have a common parent not in* *Y*. *Let* $z_x$ *and* $z_y$ *be parents of* *x* *and* *y* *not contained in* *Y*. *Neither* $(z_x,x)$ *nor* $(z_y,y)$ *is movable, so* $z_x$ *forms a triangle together with its parent* $c_x$ *and its other child* $d_x$, *and similarly* $(c_y,z_y,d_y)$ *also forms a triangle.* We distinguish three subcases:

1. $\varvec{d_y\not \in Y}$ Inverting the orientation of $(z_y,d_y)$ gives a valid orientation with the same underlying unrooted network, and conserves the downward-closedness of *Y* and the isomorphism between *Y* and $Y'$ (Fig. 20). The resulting rooted network has a reticulation node directly above *Y*, so we can add a node to *Y* and $Y'$ with at most one move.
2. $\varvec{d_x\not \in Y}$ This case is handled symmetrically to Case 1 by inverting the orientation of $(z_x,d_x)$.
3. $\varvec{d_x,d_y\in Y}$ Note that one of $(u',x')$ and $(u',y')$ is movable (Observation 2.10).
  - $\varvec{(u',x')}$ **is movable** Tail moving $(u',x')$ to an incoming edge of $d_x'$, the node in $Y'$ corresponding to $d_x$, we create a lowest tree node $z_x'$ with children $x'$ and $d_x'$ in $Y'$ (Fig. 21). We can add $z_x$ (with children *x* and $d_x$) and $z_x'$ (with children $x'$ and $d_x'$) to *Y* and $Y'$ at the cost of one move.
  - $\varvec{(u',y')}$ **is movable** This case is handled symmetrically to the previous case by interchanging the roles of *x* and *y*.

This proves that we can always add a node to *Y* and $Y'$ using at most one SPR move. Hence, the number of SPR moves needed to transform $N_r$ into $N_r'$ is at most $|V| = n+2k$. Together with the moves needed to get to $N_r$ and $N_r'$ from *N* and $N'$, we get an upper bound of $n+\frac{8}{3}k$ for the number of SPR moves needed to go from *N* to $N'$. $\square$

## Discussion

In a rooted phylogenetic tree, each rSPR move is a tail move, while in a network one can also perform head moves. Hence, it is natural to define rSPR moves in networks as the union of head and tail moves (Gambette et al. 2017). However, we have shown that to connect the tiers of phylogenetic network space, tail moves are sufficient (except for one trivial case with only two taxa). In fact, tail moves, by themselves, can also be seen as a natural generalization of rSPR moves on trees to networks.

Nevertheless, there can be several reasons to consider head moves in addition to tail moves when exploring the tiers of network space. First of all, the number of tail moves that one needs to transform one network into another may be bigger than the number of rSPR moves. The difference is not very big though; we have shown that at most four tail moves are sufficient to mimic one head move (assuming $|X|\ge 3$). Another reason to use head moves is when a search using only tail moves gets stuck in a local optimum. A head move can then be used to escape from this local optimum. Notice that a head move can basically move a reticulation to a completely different location in the network. Hence, from a biological point of view, head moves can change a network more drastically than tail moves.

On rooted phylogenetic trees, rNNI moves can be seen as distance-1 rSPR moves. Therefore, it is natural to define rNNI moves in the same way on networks (see Gambette et al. 2017). Hence, rNNI moves on networks are distance-1 head moves and distance-1 tail moves. Also for this case, we have shown that the distance-1 head moves may be omitted while preserving connectivity (again excluding one two-taxa case).

To determine which moves explore network space most efficiently, practical experiments are necessary. It seems reasonable to believe that a combination of different types of moves will work best. Distance-1 head and tail moves are local moves that change only a small part of the network, and the number of possible moves is relatively small. Therefore, such moves are suitable for finding a local optimum. General tail and head moves are not local and can therefore be useful to move away from local optima in order to search for a global optimum. Implementing and analysing such local-search methods is an important topic for future research.

**Fig. 22:**
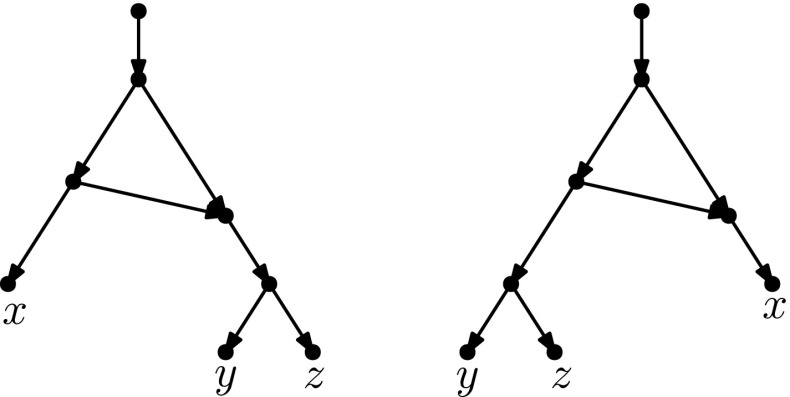
Two networks for which subtree reduction does not preserve tail-move distance

Another interesting direction is to develop algorithms for computing the tail-move distance between networks. Although we have shown that this problem is NP-hard, we do not know, for example, whether it is fixed-parameter tractable, with the tail move distance as parameter. We note that several common reduction rules, such as subtree and cluster reduction, are not always safe for this problem, in the sense that applying them may increase the tail move distance (see Bordewich and Semple 2005; Bordewich et al. 2017b, for a definition of these reductions and the chain reduction mentioned below). As an extreme example, consider the networks in Fig. 22, which have tail-move distance 3. After reducing the subtrees on $\{y,z\}$ to a single leaf, the tail move distance becomes infinite. It could, however, be safe to reduce subtrees to size 2. Similar arguments hold for cluster reduction. Another interesting question is whether reducing chains to length 2 (or any other constant length) is safe.

Finally, a major open problem concerns the rNNI diameter as well as the distance-1 tail move diameter: Are these diameters quadratic in the number of leaves, or $O(n\log (n))$? For tail, rSPR and SPR moves, we know that the diameter is linear in the number of leaves, but it would be interesting to find tight bounds (see Table 1).

## Footnotes

1 Mycorrhizal forests are so-called because of their similarity to real-life mycorrhizal networks, in which a number of trees may be connected together by an underground network of fungi.
